# Diabetes self-management education programs: Results from a nationwide population-based study on characteristics of participants, rating of programs and reasons for non-participation

**DOI:** 10.1371/journal.pone.0310338

**Published:** 2024-09-12

**Authors:** Solveig Weise, Yong Du, Christin Heidemann, Jens Baumert, Thomas Frese, Marcus Heise

**Affiliations:** 1 Institute of General Practice and Family Medicine, Medical Faculty, Martin-Luther-University Halle-Wittenberg, Halle (Saale), Saxony-Anhalt, Germany; 2 Department of Epidemiology and Health Monitoring, Robert Koch Institute, Unit Physical Health, Berlin, Berlin, Germany; University of Benghazi, LIBYA

## Abstract

**Objective:**

Population-based studies of reasons for not participating in diabetes self-management education (DSME) are scarce. Therefore, we investigated what sociodemographic and disease-related factors are associated with participation in DSME, the reasons for not participating in DSME and how participants evaluate DSME.

**Research design and methods:**

We used data from the nationwide survey “Disease knowledge and information needs–Diabetes mellitus 2017”, which included a total of 1396 participants diagnosed with diabetes mellitus (diabetes; n = 394 DSME-participants, n = 1002 DSME-never-participants). Analyses used weighted logistic or multinominal regression analyses with bivariate and multivariable approaches.

**Results:**

Participants were more likely to attend DSME if they had a medium (OR 1.82 [95%CI 1.21–2.73]),or high (OR 2.04 [95%CI 1.30–3.21]) level of education, had type 1 diabetes (OR 2.46 [1.24–4.90]) and insulin treatment (OR 1.96 [95%CI 1.33–2.90]). Participants were less likely to attend DSME if they lived in East Germany (OR 0.57 [95%CI 0.39–0.83]), had diabetes for >2 to 5 years (OR 0.52 [95%CI 0.31–0.88] compared to >5 years), did not agree that diabetes is a lifelong disease (OR 0.30 [95%CI 0.15–0.62], had never been encouraged by their physician to attend DSME (OR 0.19 [95%CI 0.13–0.27]) and were not familiar with disease management programs (OR 0.67 [95%CI 0.47–0.96]). The main reasons for non-participation were participant’s personal perception that DSME was not necessary (26.6%), followed by lack of recommendation from treating physician (25.7%) and lack of information on DSME (20.7%). DSME-participants found DSME more helpful if they had a medium educational level (OR 2.06 [95%CI 1.10–3.89] ref: low level of education) and less helpful if they were never encouraged by their treatment team (OR 0.46 [95%CI 0.26–0.82]).

**Discussion:**

Professionals treating persons with diabetes should encourage their patients to attend DSME and underline that diabetes is a lifelong disease. Overall, the majority of DSME participants rated DSME as helpful.

## Introduction

Diabetes mellitus (diabetes) is a global public health issue [1]. To control blood sugar effectively and minimize long-term complications, diabetes necessitates comprehensive diabetes self-management education (DSME) as a vital component of care [2, 3]. DSME significantly enhances self-management, contributing to better metabolic control [4]. In Germany, DSME is mostly provided as group-based education and is usually provided by specially trained nurses, while general practitioners (GPs) or diabetologists typically provide a single session of DSME [5]. Persons with type 2 diabetes usually receive four weekly 90-minute DSME sessions, extendable to 20 hours for higher-risk patients. Persons with type 1 diabetes undergo extended DSME, spanning multiple days. Research consistently shows that DSME participants exhibit improved lifestyle choices and enhanced self-management behaviors, even in routine care settings [6, 7]. Furthermore, RCTs demonstrated reduced mortality in DSME participants [8].

Despite the significant benefits of DSME, a notable proportion of persons with diabetes (PWD) do not participate in DSME [9]. A German population-based study found that 37.3% did not attend DSME, despite the fact that the German Statutory Health Insurance fully reimburses attendance [6]. In the UK, 90% of invited PWD did not attend DSME [10]. This is concerning as DSME non-participants have been shown to have lower adherence to self-management behaviors and secondary preventive exams such as retinopathy screening and medical foot examination [7]. Understanding the reasons for non-participation is crucial. It enables policymakers and healthcare professionals (HCP) to effectively motivate PWD to engage in DSME [11]. Previous studies revealed logistical, medical or financial reasons as barriers for participation [9, 12, 13]. However, all studies were limited to a small sample size [9]. In Germany, DSME participation is available through nationwide outpatient care within the diabetes disease management program (DMP), fully covered by statutory health insurance.

This paper addresses the following research questions: 1) What are the sociodemographic and disease-related factors associated with participation in DSME? 2) What are the main reasons to decline participation in DSME? 3) How do participants evaluate DSME programs, and does evaluation vary with participant characteristics?

## Materials and methods

### Sample

We used data from the nationwide “Disease knowledge and information needs–Diabetes mellitus 2017”-survey, conducted between September and November 2017 in Germany. The survey was designed by the Robert Koch Institute in cooperation with the Federal Centre for Health Education (BZgA) and the Institute of Medical Sociology and Rehabilitation Science of the Charité-Universitätsmedizin Berlin. The data stemmed from computer-assisted telephone interviews and due to its dual-frame design can be considered as representative of all private households in Germany that can potentially be reached by telephone. Details on the composition of the sample and the implementation of the survey are described elsewhere [14, 15].

Out of 1479 survey participants with diabetes, we considered 1396 for the first research question, including those with recent diabetes (n = 1386) and those currently on antidiabetic medications (n = 10). For the second research question, we examined a subset of 394 participants who had never received DSME. For the third research question, we analyzed a subgroup of 1002 DSME participants.

### Ethics approval

The study was approved by the ethics committee of Berlin’s Chamber of Physicians (reference number: Eth-23/17) and the Federal Commissioner for Data Protection and Freedom of Information of Germany. The study was performed according with the principles of the Declaration of Helsinki [16]. All interviewees provided verbal consent at the start of the phone interview, having been briefed on the study’s purpose, data protection, and their voluntary participation.

### Assessment of diabetes

Participants were asked if they had ever been diagnosed with diabetes by a physician. Those who answered affirmatively were asked whether diabetes had persisted in the past 12 months and whether they were currently undergoing treatment through diabetes medication, lifestyle (diet or physical activity). Participants who reported having diabetes in the past 12 months or being treated with antidiabetic medication were classified as having diabetes.

### Outcomes

The outcomes of our analysis included DSME participation, reasons for non-participation in DSME, and perceived benefits of DSME.

PWD were asked whether they had ever attended a DSME and whether it was a group or an individual DSME. Respondents who answered yes to at least one statement were classified as DSME participants, while respondents who answered no to both questions were classified as never-DSME participants.

Never-DSME participants were queried about their primary reason for not participating in DSME and could select from eight predefined response categories. DSME participants were asked to retrospectively rate the perceived usefulness of DSME on a four-point Likert scale (“Overall, how helpful was this training in helping you manage your diabetes better?”) [17]. Following the DAWN2 Study Group’s analysis [17], we categorized this variable as "somewhat/very helpful" vs. "not at all/rather less helpful.

### Socio-demographic characteristics

The following socio-demographic characteristics were included in the statistical analyses: Age (categorized as 18 to 64 years/65 to 79 years/≥80 years), sex (male/female), living situation (alone/living together with partner), educational level (classified according to CASMIN [18]), occupational status (employed/not employed including students, homemakers, retired or disabled respondents) and residency (West Germany/East Germany).

### Disease-related characteristics

The following disease-related characteristics were included in the statistical analyses: Self-reported type of diabetes (type 1/type 2), time since diagnosis of diabetes mellitus (≤2 years / > 2 to 5 years/>5 years), current therapy with insulin (yes/no), non-insulin medication (oral antidiabetics or other blood glucose-lowering medications than insulin that are injected; yes/no), and lifestyle-therapy (physical activity or diet; yes/no).

### Beliefs and information about diabetes

Regarding beliefs and information about diabetes, we included an item from the “optimistic bias” subscale of the Risk Perception Survey-Diabetes Mellitus [19]. It stated “Compared to other people with diabetes of my age and gender, I have a lower risk of developing diabetes complications.” (fully or rather agree/fully or rather disagree).

From the German version of the Revised Illness Perception Questionnaire (IPQ-R) we adopted the subscale “personal control” [20, 21]. This subscale consists of four items on a Likert scale that form a summative index ranging from 4 to 20 points, with higher scores indicating a greater degree of control beliefs over one’s illness. Within the present sample, the index had an internal consistency of Cronbach’s α = 0.76.

Also, from the IPQ-R, we adopted a single item from the “timeline acute/chronic” subscale, worded: “I expect to have diabetes for the rest of my life” (fully or rather agree/fully or rather disagree or undecided).

From a Dutch survey [22] investigating the perceived risk for type 2 diabetes, we adopted the item “I consider diabetes to be a serious disease.” (severe or very severe disease/not or somewhat severe disease or no opinion).

Following an item from the DAWN2 study [23], respondents were asked if they were encouraged by their HCP to attend a specific group or training to help them manage their diabetes (never/rarely to always).

Finally, respondents were asked to indicate if they were familiar with the disease management program (DMP) for PWD (yes/no).

### Statistical analysis

For the three aforementioned outcomes, we computed unweighted absolute frequencies and weighted relative frequencies, along with their respective 95% confidence intervals (95% CIs). In bivariate analyses, these frequencies were stratified by sociodemographic factors, disease-related characteristics, as well as beliefs and information about diabetes. Considering the survey design and the applied weighting procedure, we employed the Rao and Scott correction on the Pearson-χ^2^ statistic [24] to assess the statistical significance of bivariate associations between exogenous factors and categorical outcomes.

For the two dichotomous outcomes “DSME-participation” and “perceived benefit of DSME”, partial effects of exogenous factors were determined in weighted (binomial) logistic regression analyses. For the categorical endpoint “reasons for not participating in DSME”, we used multinomial logistic regression. In our regression analyses, we chose independent variables based on the criteria outlined by Hosmer et al. [25]. Initially, all variables with an univariable p-value < 0.25 were included. Subsequently, we employed a backward stepwise selection approach, excluding independent variables with a Wald statistic p-value > 0.05 to create the final model. This process led to distinct sets of independent variables for the three outcomes. The results were presented with odds ratios or relative risk ratios, accompanied by their respective 95% CIs.

We weighted all relative frequencies, odds ratios and relative-risk ratios with respect to gender, age, education, and state. This procedure accounted for possible deviations between the sample of PWD in the present study and the reference population with diabetes from the German Health Update (GEDA) 2012 study. Further details of the weighting procedure are described elsewhere [15]. In order to address missing data, we applied multiple imputations by chained equations (m = 10) [26] within a sensitivity analysis for all multiple regression models. Implementing fully conditional specifications [27], all model-immanent factors served as auxiliary variables. We set the two-sided significance level at 5%. We performed all analyses using STATA version 16.1 (Stata Corp., College Station, TX).

## Results

**Fig 1:**
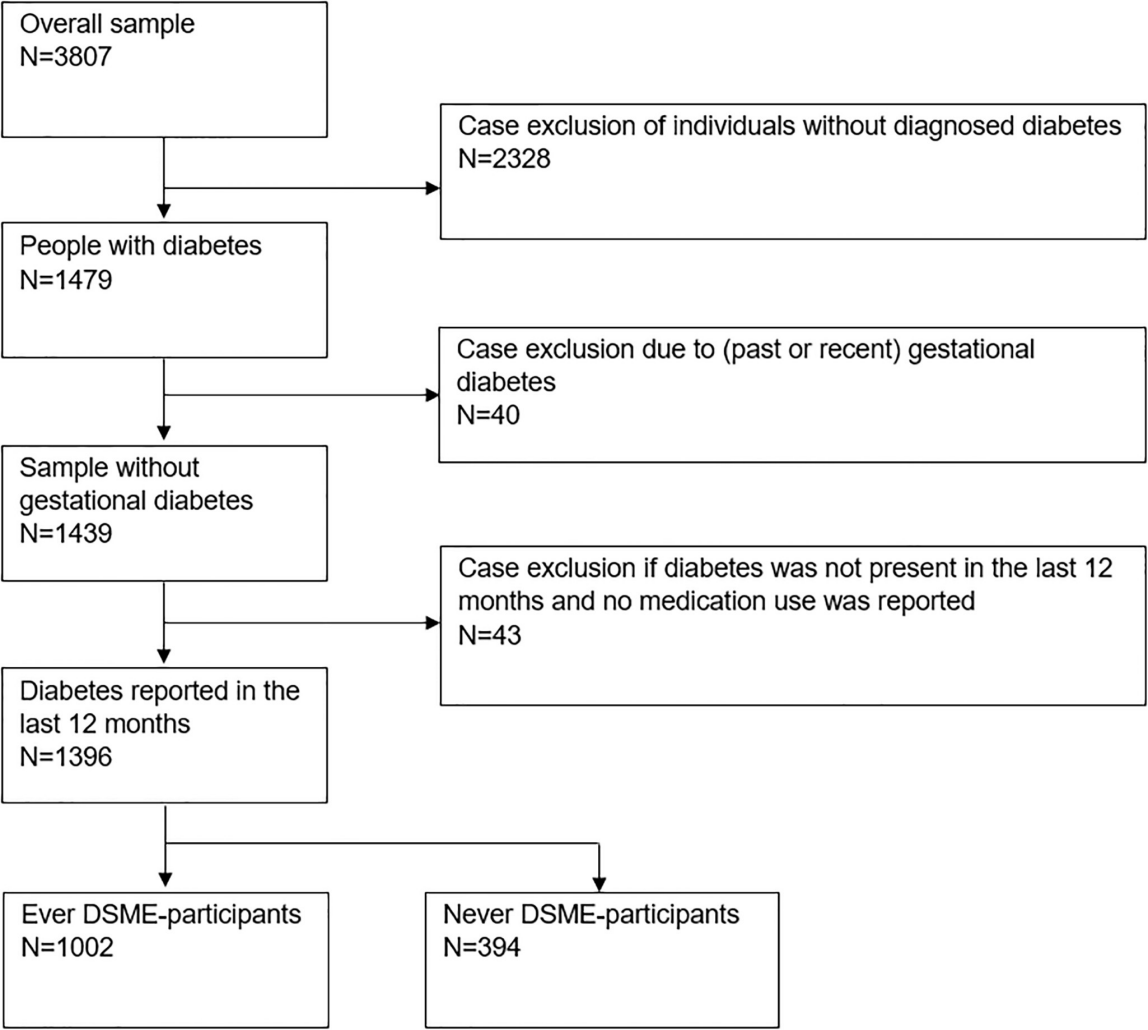
Flow chart of study participants. Abbreviations: DSME–structured diabetes self-management education.

Of the 1396 study PWD, 1002 (73.0%) were ever-DSME participants and 394 (27.0%) never-DSME participants (Table 1, Flow-chart in Fig 1).

**Table 1:** Absolute and weighted relative frequencies of respondents who ever participated in DSME training, stratified by socio-demographic characteristics, disease-related characteristics and beliefs about diabetes.

	participants / n_valid_	weighted relative frequency		Test for difference
		f	95% C.I.	p
**Overall (n = 1396)**	1002 / 1396	73.0%	[70.0%; 75.8%]	
**Socio-demographic characteristics**				
**Age (n = 1396)**				**p < 0.01**
18 to 64 years	342 / 430	78.9%	[73.5%; 83.5%]	
65 to 79 years	493 / 685	72.1%	[68.0%; 75.9%]	
over 80 years	167 / 281	57.8%	[50.9%; 64.4%]	
**Sex (n = 1396)**				p = 0.223
Male	527 / 719	74.8%	[70.7%; 78.5%]	
Female	475 / 677	71.2%	[66.7%; 75.3%]	
**Living situation (n = 1394)**				p = 0.411
Living alone	425 / 612	71.7%	[67.0%; 76.0%]	
Living together with partner	576 / 782	74.2%	[70.2%; 77.8%]	
**Educational level (n = 1394)**				p = 0.098
Low	269 / 395	70.0%	[64.6%; 74.9%]	
Middle	437 / 594	75.8%	[71.9%; 79.4%]	
High	295 / 405	75.2%	[69.7%; 79.9%]	
**Occupational status (n = 1394)**				**p = 0.014**
Not employed *	783 / 1121	70.4%	[67.0%; 73.6%]	
Employed	217 / 273	80.0%	[73.1%; 85.5%]	
**Residency (n = 1396)**				p = 0.054
West Germany	659 / 885	74.9%	[71.2%; 78.4%]	
East Germany	343 / 511	69.0%	[63.8%; 73.7%]	
**Disease-related characteristics**				
**Type of Diabetes (n = 1316)**				**p < 0.01**
Type 1 diabetes	151 / 167	90.6%	[83.6%; 94.8%]	
Type 2 diabetes	802 / 1149	70.4%	[67.0%; 73.7%]	
**Time since diagnosis (n = 1388)**				**p < 0.01**
2 years or less	43 / 86	49.4%	[37.3%; 61.6%]	
> 2 years to 5 years	114 / 185	63.6%	[53.7%; 72.4%]	
More than 5 years	843 / 1117	77.2%	[74.0%; 80.0%]	
**Non-insulin medication (n = 1396)**				**p = 0.013**
Currently not administered	391 / 499	78.0%	[72.8%; 82.5%]	
Current therapy	611 / 897	70.1%	[66.3%; 73.6%]	
**Insulin (n = 1395)**				**p < 0.01**
Currently not administered	443 / 730	64.0%	[59.8%; 68.0%]	
Current therapy	558 / 665	81.9%	[77.3%; 85.7%]	
**Lifestyle therapy (n = 1396)**				p = 0.107
Currently not administered	254 / 385	69.4%	[63.9%; 74.4%]	
Physical activity and/or dietary therapy	748 / 1011	74.5%	[70.8%; 77.8%]	
**Beliefs and information about diabetes**				
**Low perceived risk of diabetes complications (n = 1232)**				p = 0.286
(Fully / rather) agreement	499 / 698	71.9%	[67.8%; 75.6%]	
(Fully / rather) disagreement	394 / 534	75.4%	[70.1%; 80.0%]	
**Personal control subscale (IPQ-R) (n = 1315)**				**p = 0.034**
High (above median of 16)	378 / 509	77.0%	[72.4%; 81.0%]	
Low (equal/below median of 16)	570 / 806	70.5%	[66.2%; 74.4%]	
**“I suppose I will have diabetes for the rest of my life” (n = 1386)**				**p < 0.01**
(Fully / rather) agreement	941 / 1286	74.8%	[71.8%; 77.6%]	
Does not agree (at all) / undecided	55 / 100	55.2%	[41.3%; 68.4%]	
**“I consider diabetes to be a serious disease” (n = 1386)**				**p = 0.019**
(Very) severe disease	552 / 732	76.2%	[72.1%; 79.8%]	
Not / somewhat severe / no opinion	443 / 654	69.1%	[64.4%; 73.4%]	
**Healthcare team encouraged to attend any group or training (n = 1384)**				**p < 0.01**
Rarely to always	606 / 697	86.3%	[82.5%; 89.4%]	
Never	387 / 687	56.2%	[51.6%; 60.6%]	
**“Are you familiar with DMP?” (n = 1391)**				**p < 0.01**
Yes	550 / 697	79.4%	[75.3%; 83.0%]	
No	447 / 694	66.5%	[61.9%; 70.7%]	

* The category “not employed” includes students and homemakers as well as retired or disabled respondents; Abbreviations: DMP–Disease-Management-Programme; DSME–structured diabetes self-management education; IPQ-R–Revised Illness Perception Questionnaire-subscale for control belief

### Which sociodemographic and disease-related factors are associated with participation in DSME?

In bivariate analyses (Table 1), PWD aged over 80 years (p < 0.01) and unemployed PWD (p = 0.014) were significantly less likely to participate in DSME. Type 1 diabetes (p < 0.01), time since diagnosis longer than five years (p < 0.01) and insulin treatment (p < 0.01) were positively associated with DSME participation. Treatment with non-insulin medication (p = 0.013) was negatively associated. Low personal control beliefs (p = 0.034) and the belief, that diabetes is neither a lifelong (p < 0.01) nor serious (p = 0.019) illness, were significantly associated to low participation rates in DSME. Familiarity with the DMP (p < 0.01) and encouragement from the HCP (p < 0.01) were significantly positively associated with DSME participation in bivariate analyses. S1 Table gives a comparison of these characteristics between DSME-participants and never-DSME-participants.

**Fig 2:**
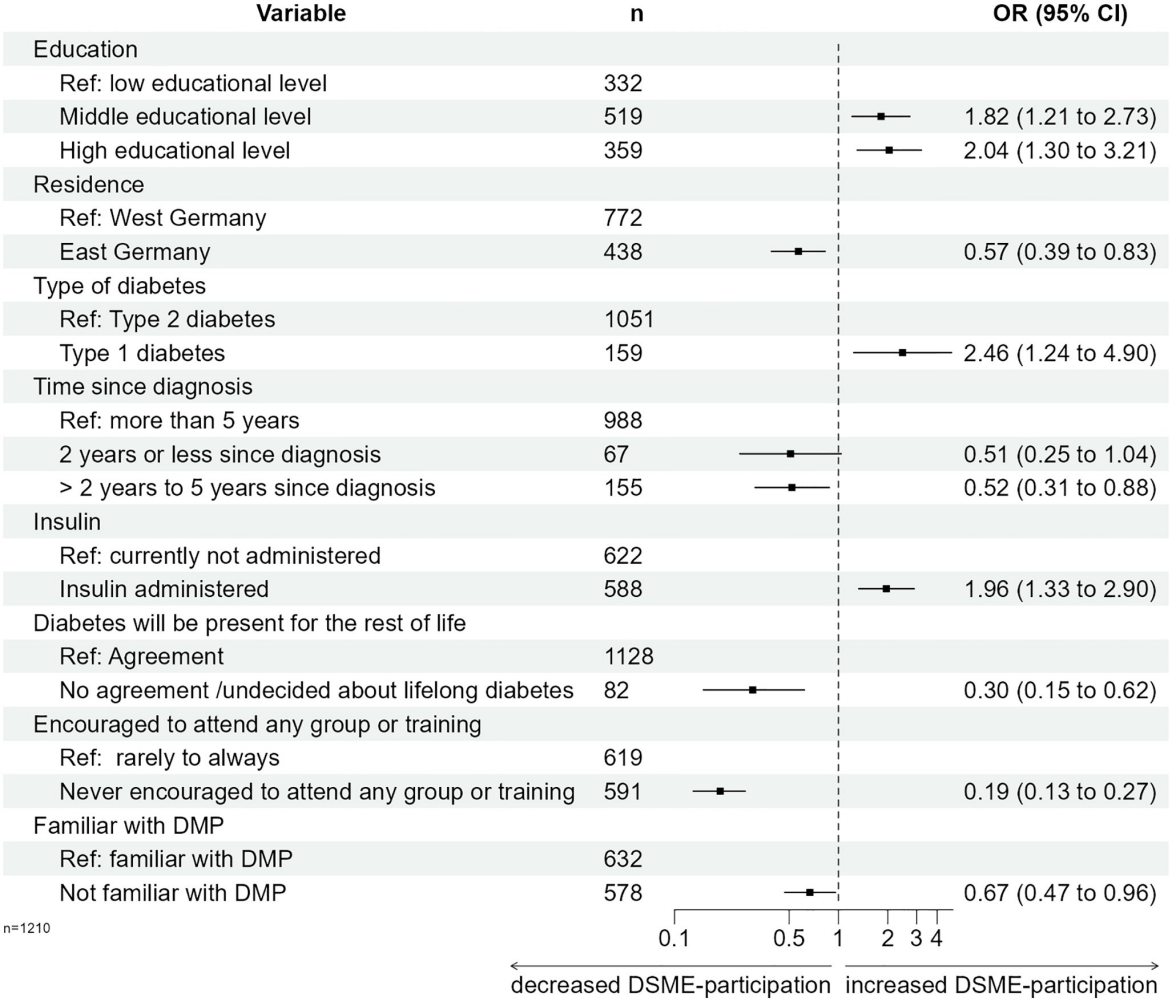
Final model of weighted logistic regression of DSME-participation on socio-demographic and disease-related characteristics, beliefs and information about diabetes (complete case analysis for n = 1210). Abbreviations: DMP–Disease-Management-Programme; DSME–structured diabetes self-management education.

The final multivariable logistic regression model (Fig 2 and S2 Table) confirmed that PWD who had never been encouraged by their HCP to attend any DSME (OR = 0.19; p < 0.001) and respondents unfamiliar with DMPs (OR = 0.67; p = 0.029) participated significantly less frequently in DSME. Disagreeing with the statement "diabetes is a lifelong disease" was linked to lower DSME participation (OR = 0.30; p = 0.001). Respondents with a diabetes diagnosis of more than two to five years (compared to more than five years) had a significantly lower likelihood of participating in DSME (OR = 0.52; p = 0.014). In contrast, respondents with type 1 diabetes (OR = 2.46; p = 0.010) and respondents receiving insulin therapy (OR = 1.96; p = 0.001) participated more frequently in DSME than those with type 2 diabetes and without insulin therapy. A medium (OR = 1.82; p = 0.004) and a high (OR = 2.04; p = 0.002) educational level were positively associated with DSME participation compared to low educational level. PWD in East Germany were less likely to participate in DSME than PWD residing in West Germany when controlling for other model-immanent variables (OR = 0.57; p = 0.003). The concordance statistic of both regression models (c = 0.796 and 0.790) indicated that the DSME-participation varies considerably depending on participant-related characteristics. The results of the multiple imputation sensitivity analysis were consistent with these findings (S3 Table).

### What are the main reasons to decline participation in DSME?

Univariate analysis revealed that 48.6% of never-DSME participants cited lack of information or recommendation as the main reason for not participating in DSME (Table 2). Among these respondents, 25.7% indicated that their physician did not recommend DSME and 20.7% felt insufficiently informed about DSME. Among PWD stating other reasons for not participating in DSME (51.4%), most indicated that they personally did not find it necessary (26.6%). Subsequent bivariate analyses are shown in S4 Table.

**Table 2:** Main reason for not participating in DSME, absolute and weighted relative frequencies (never-DSME participants, n = 394).

	n /n_valid_	f	95% C.I.
**Reasons related to lack of information or recommendation**	193 / 389	48.6%	[42.3%; 55.0%]
Because I feel too little informed about the programme.	9 / 389	2.2%	[1.1%; 4.4%]
Because my treating physician did not recommend it.	103 / 389	25.7%	[20.2%; 32.1%]
Programmes were or are not known to me.	81 / 389	20.7%	[16.3%; 26.0%]
**Further reasons for not participating in DSME**	196 / 389	51.4%	[45.0%; 57.7%]
Because my illness does not allow it/because I feel too ill.	16 / 389	4.8%	[2.8%; 8.2%]
Because I personally do not find it necessary.	111 / 389	26.6%	[21.7%; 32.1%]
Because I had no time / too much effort.	23 / 389	6.1%	[3.8%; 9.4%]
Because I do not think, it will help me.	8 / 389	3.1%	[0.9%; 10.0%]
Other reasons (not specified)	38 / 389	10.8%	[7.5%; 15.3%]
Don’t know / refused to answer	5		

Abbreviations’–structured diabetes self-management education; n–absolute frequencies, n_valid_−number of participants with valid responses

**Fig 3:**
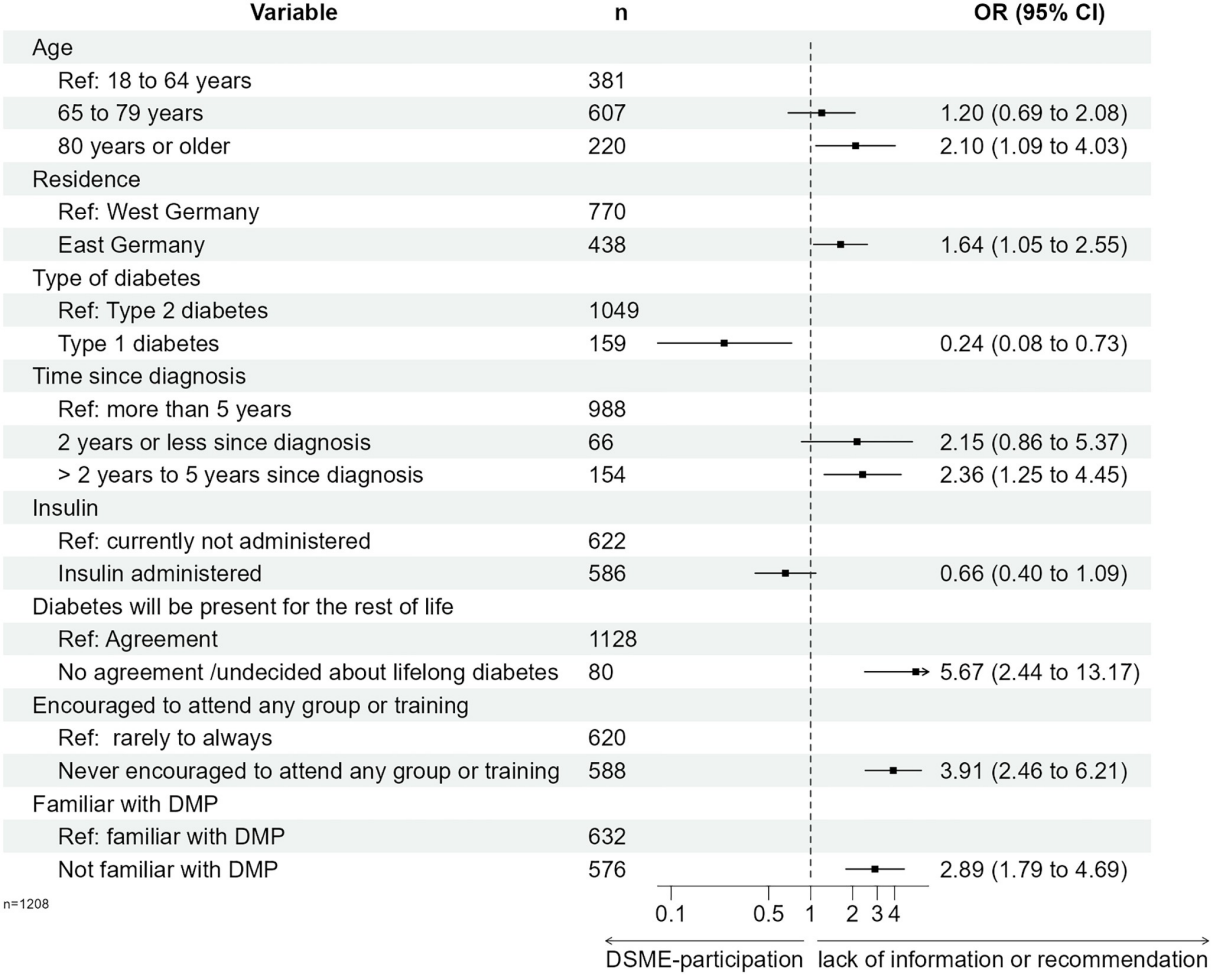
Weighted multinominal regression for lack of information or recommendation as main reason for not participating in DSME. Abbreviations: DMP–Disease-Management-Programme; DSME–structured diabetes self-management education.

**Fig 4:**
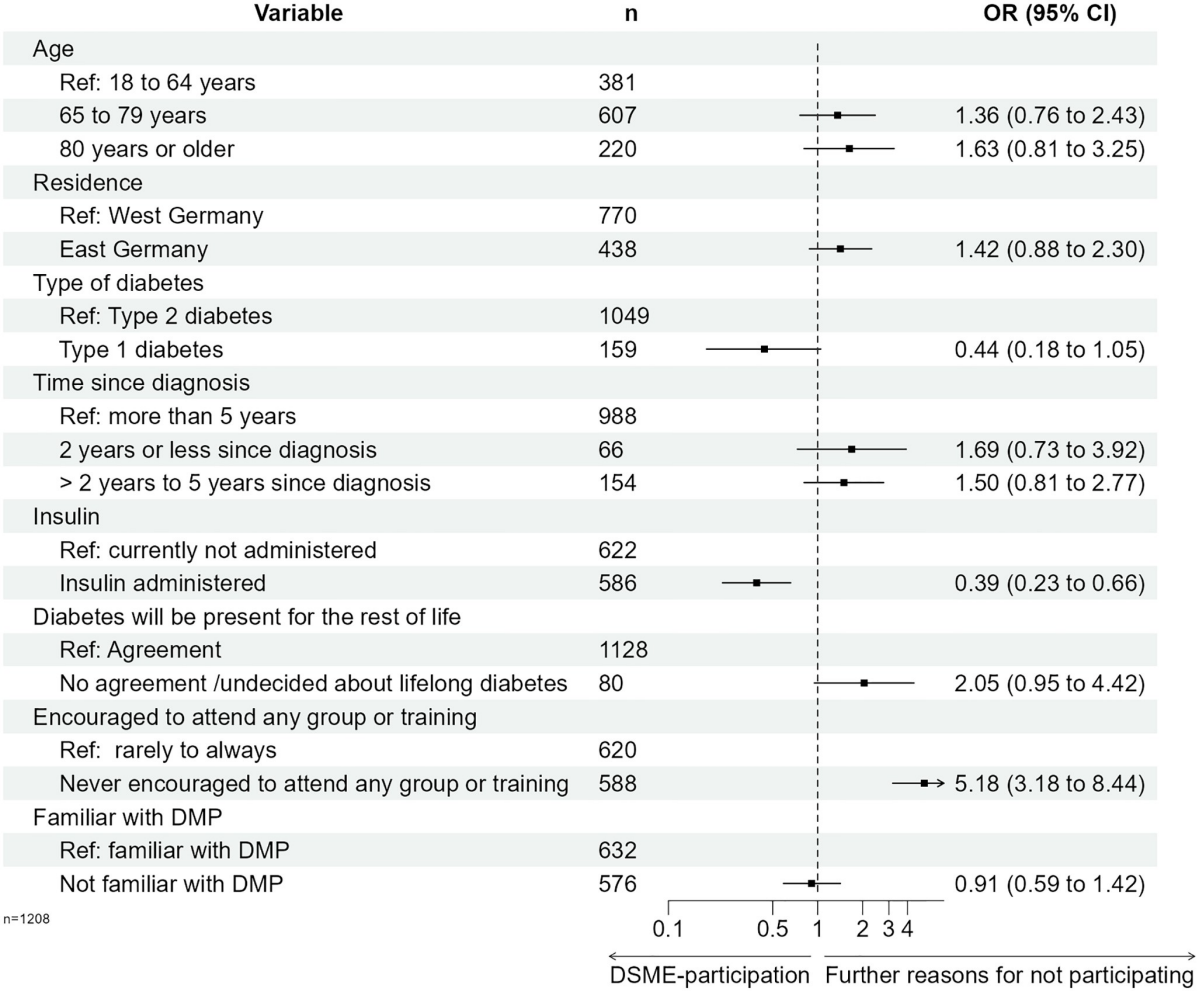
Weighted multinominal regression for further reasons for not participating in DSME. Abbreviations: DMP–Disease-Management-Programme; DSME–structured diabetes self-management education.

Fig 3 and S5 Table show the results of a weighted multinomial logistic regression for the main reason to decline DSME-participation. Compared to DSME participants, never-DSME participants who identified a lack of information or recommendations as the main reason for non-participation were significantly more likely to be older than 80 years (RRR = 2.10; p = 0.026), reside in East Germany (RRR = 1.64; p = 0.029), and have received a diabetes diagnosis between two and five years ago (RRR = 2.36; p = 0.008). Furthermore, participants who reported a lack of information or recommendation were more frequently unfamiliar with DMP (RRR = 2.89; p < 0.001), had never been encouraged by their HCP to attend DSME (RRR = 3.91; p < 0.001) and more frequently neglected that diabetes were a lifelong disease (RRR = 5.67; p < 0.001). PWD with type 1 diabetes less frequently reported a lack of information or recommendation (RRR = 0.24; p = 0.012; S5 and S6 Tables, Fig 3). Further reasons for not participating in DSME were more common among respondents who were never encouraged by their HCP (RRR = 5.18; p < 0.001) and less common among respondents with insulin therapy (RRR = 0.39; p < 0.001, Fig 4, S5 and S6 Tables). The results of the sensitivity analysis are consistent with these findings (S7 Table).

### How do participants assess DSME programs, and is there a variation in evaluation based on participant characteristics?

Bivariate analyses in S8 Table showed that male PWD generally attributed greater benefits to DMSE programs than female (p = 0.046). For disease-related factors, we found that PWD who were currently receiving lifestyle therapy rated DSME as significantly better compared to respondents not currently engaged in lifestyle therapy (p = 0.042). In addition, never having been encouraged by the HCP to attend any form of training was negatively associated with the perceived usefulness of DSME (p < 0.01).

**Fig 5:**
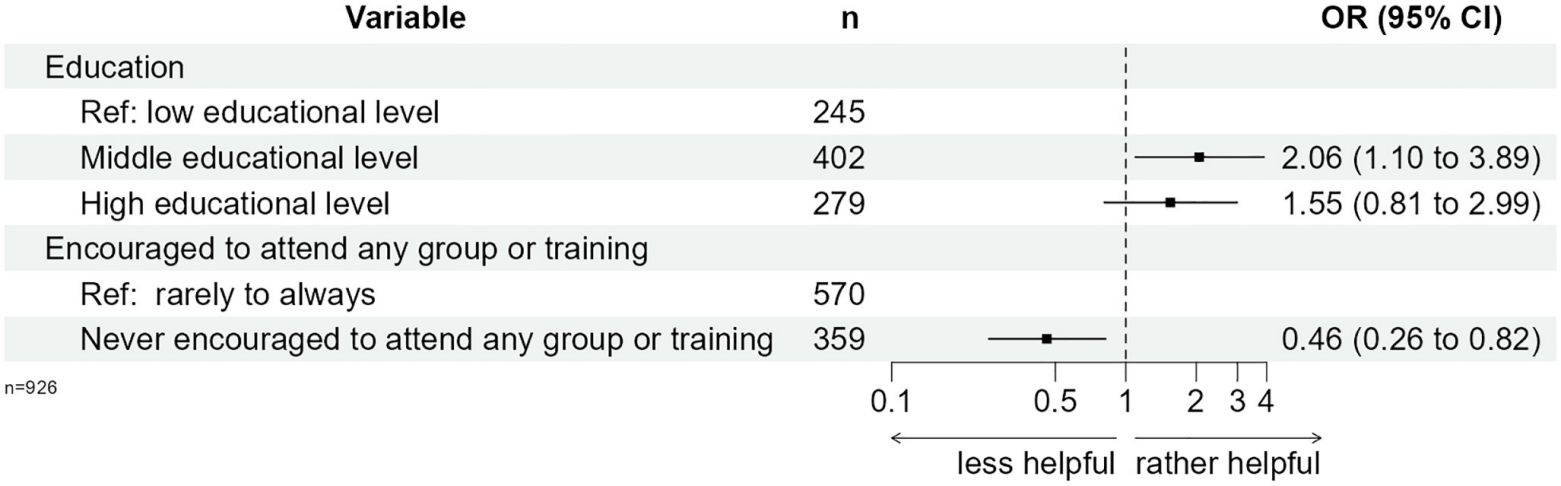
Weighted logistic regression of perceived benefit of DSME (“somewhat / very helpful” vs. “not at all / rather less helpful”).

Within multivariable logistic regression analyses, medium educational level was positively associated with the perceived benefit of DSME (OR = 2.06; p = 0.025; Fig 5, S9 Table). Never being encouraged to attend DSME by the HCP remained a significant factor of the negative perceived usefulness of DSME (OR = 0.46; p = 0.008; Fig 5, S9 Table). The results of the sensitivity analysis are in agreement with these findings (S10 Table).

Individual DSME and group DSME were not perceived as significantly different by PWD (S11 Table). On average, PWD assessed both DSME formats as “somewhat helpful” (32.8% of valid responses) or “very helpful” (57.4% of valid responses).

## Discussion

### Main findings

In this nationwide cross-sectional population-based study, we found that PWD were significantly more likely to participate in DSME if they had a medium or high level of education, had type 1 diabetes, were receiving insulin treatment, and had diabetes for more than five years. PWD were significantly less likely to participate in DSME if they did not consider diabetes to be a lifelong disease, had never been encouraged by HCP to participate in DSME, or were unfamiliar with DMPs. The main reasons for not attending DSME were lack of information about DSME, no recommendation from the treating physician, and personally believing that DSME-participation was unnecessary. 90% of DSME participants rated DSME as very or rather helpful. Those PWD who were encouraged to participate in DSME by their HCP or had a higher educational level rated DSME as significantly more helpful. To the best of our knowledge, a nationwide analysis of reasons for non-participation in DSME and relationship between patients’ perceived helpfulness of DSME and HCP support has not yet been published.

### Comparison with other studies

This analysis identified a DSME participation rate higher than what has been reported in previous literature. A population-based study from Germany reported a DSME-participation rate of 62.7% [6], while a study of a US sample reported an attendance rate of 53.7% [28]. These differences in DSME-participation rates may be attributed to variations in health systems, study design or time of data collection. As DSME is fully reimbursed by the German statutory health insurance system, it is available to almost all PWD. Previous studies have examined reasons for non-participation in DSME [9, 12, 29–34], but few, like our study, have examined DSME participation in routine care [34]. Given the scarcity of population-based, nationwide data on DSME participation and individual motivation to decline DSME, the present analyses address an important issue.

Our results underline findings from previous studies concerning reasons for DSME non-participation [9, 12, 29–34], lack of referral to DSME or lack of information about DSME, respectively [9, 12, 30, 33] and limited knowledge about diabetes being a lifelong disease [32]. However, the latter theme was less prominent in our study. Our data shows that one-quarter of never-DSME participants were not encouraged to attend DSME by their treating physician. A recent study shows that the treating HCP are the main source of information for most PWD [12]. Our results also showed that recommendations and referrals to DSME were associated with DSME perceived benefit. This underscores the importance of explicit recommendations and referrals to DSME by HCP. To our knowledge, this relationship between patients’ perceived helpfulness of DSME and HCP support has not been investigated before.

Our study showed that unfamiliarity with the DMP was a significant barrier to DSME participation. If the registered DMP patients correlate with documented type 1 and type 2 diabetes cases, DMP participation rates are 63% and 58%, respectively [35]. GPs receive an extra payment for enrolling patients in DMPs. However, if they refer their patient to the DSME and the PWD does not participate within six months, the PWD is excluded from the DMP and its GP loses this extra payment. This may be a barrier for DSME recommendation for some GPs in Germany. Internationally, other barriers for GP referrals to DSME include concerns about care fragmentation, uncertainty about the GP’s role in diabetes care, and the need for improved motivational skills training [36]. These barriers have not been examined in our study.

PWD living in East Germany were significantly less likely to participate in DSME. Diabetes prevalence is significantly higher in East than in West Germany [37]. Eastern Germany is socioeconomically more deprived with a lower physician density. This may be an explanation of our findings.

We showed that PWD with a low educational level or an age of 80 and above, respectively, participated significantly less often in DSME. This is in concordance with international literature [12, 33]. Due to the less strict HbA1c targets in older age and reduced mobility, physicians might not feel inclined to inform and refer older PWD to DSME [3, 9]. As for PWD with moderate to severe illness or an age of 80 and above, DSME participation may be an individual decision. This decision should be made via the shared-decision process between PWD and physican and take into account if the individual PWD really benefits from a DSME participation.

In our sample, a higher education level was positively associated with both DSME participation and how participants perceived its benefits. Previous studies showed that DSME mainly addresses instructor-led and application-oriented learning types [38]. This calls for DSME programs to be adapted to the needs of individual patients.

Literature had already called for the necessity to promote and market all aspects of DSME [9]. When non-attenders have been informed about DSME, many desired more information or were willing to participate [34]. Promoting the positive effects of DSME on diabetes outcomes to HCP in the hope that they will increase referrals to DSME seems logical. Time and financial restrictions may not be overcome easily. Our results show that a lack of information constitutes such an important barrier for PWD. Therefore, it appears that DSME would benefit from a major public information campaign targeting people with diabetes. Similarly examples for other preventive interventions exist as e.g. vaccination programs or cancer screenings [12, 39]. This is another topic for future studies, as these interventions need to be scientifically evaluated.

### Strengths

To the best of our knowledge, this is the first nationwide population-based study to investigate the reasons, socio-demographic and disease-related variables for non-participation in DSME. Although quantitative investigations on barriers towards DSME have been published before, [9, 30, 33, 34], most studies focused on regional data bases obtained in big cities [30, 33], a single federal state [31] or were based on a singular insurance company [34]. Our study sample includes PWD across the adult age spectrum and information on various socio-demographic, reasons for non-participation and illness-beliefs which in seldomly found in this combination.

### Limitations

Due to the cross-sectional design of our study, we cannot assume direct causality. Therefore, „lack of knowledge about DSME”and „not agreeing that diabetes is a life long illness”may not be causes for non-attendance in DSME. All included outcome and confounding variables were self-reported. Social desirability and recall errors may bias our data, especially regarding reasons for not participating in the DSME. By reporting that the treating HCP did not recommend or inform them about DSME, participants may be trying to hide other reasons for not attending. Our survey did not include variables as e.g. distance to DSME location, different timings of DSME etc. which may be included in future studies. However, addressing those variables has not been shown to increase DSME uptake [9]. Lastly, our study lacks data on HbA1c or blood glucose measures, so we cannot investigate the effect of DSME attendance or reasons for non-attendance towards HbA1c levels.

## Conclusions

In this population-based nationwide study, we showed, that PWD were significantly more likely to participate in DSME if they had a medium or high level of education, had type 1 diabetes, were receiving insulin treatment, and had diabetes for more than five years. PWD were significantly less likely to participate in DSME if they did not consider diabetes to be a lifelong disease, had never been encouraged by HCP to participate in DSME, or were unfamiliar with DMPs. The main reasons for not attending DSME were lack of information about DSME and personally beliefs that DSME-participation was unnecessary. Our findings suggest, that HCP working with PWD should focus on two main issues when promoting DSME paticipation: (1) informing and recommending DSME participation to PWD, specifically those having a lower educational level and, (2) educating their PWD that diabetes is a life-long and severe illness.

Our data and previous studies suggest that measures beyond addressing the “good will” of HCP may be necessary to promote and increase DSME attendance rates successfully. For instance, these might involve government or health insurance information campaigns modeled after vaccination or cancer screening initiatives. Clearly, future research should investigate whether these approaches are successful.

## Supporting information

S1 Table Absolute and weighted relative frequencies of respondents who ever participated in DSME training and never-participants, stratified by sociodemographic characteristics, disease-related characteristics and beliefs about diabetes. *The category “not employed” includes students and homemakers as well as retired or disabled respondents; Abbreviations: DMP–Disease-Management-Programme; DSME–structured diabetes self-management education; IPQ-R–Revised Illness Perception Questionnaire-subscale for control belief.
(DOCX)

S2 Table Weighted logistic regression of DSME-participation on socio-demographic and disease-related characteristics, beliefs and information about diabetes (complete case analysis for n = 1210). *The category “not employed” includes students and homemakers as well as retired or disabled respondents; Abbreviations: DMP–Disease-Management-Programme; DSME–structured diabetes self-management education; IPQ-R–Revised Illness Perception Questionnaire-subscale for control belief.
(DOCX)

S3 Table Sensitivity analysis for weighted logistic regression of DSME-participation on socio-demographic and disease-related characteristics, beliefs and information about diabetes (n = 1396; multiple imputation by chained equations*). * The proportion of missing information per variable ranged from 0% to 5.7%. For 13.3% of respondents, at least one value was imputed. Abbreviations: DMP–Disease-Management-Programme; DSME–structured diabetes self-management education, OR–Odds ratio.
(DOCX)

S4 Table Absolute and weighted relative frequencies of respondents who ever participated in DSME training and never-participants, stratified by socio-demographic characteristics, disease-related characteristics and beliefs about diabetes. * The category “not employed” includes students and homemakers as well as retired or disabled respondents; Abbreviations: DMP–Disease-Management-Programme; DSME–structured diabetes self-management education; IPQ-R–Revised Illness Perception Questionnaire-subscale for control belief.
(DOCX)

S5 Table Weighted multinomial logistic regression of main reason for not participating in DSME on socio-demographic and disease-related characteristics, beliefs and information about diabetes (complete case analysis for n = 1208; only final model). Abbreviations: DMP–Disease-Management-Programme; DSME–structured diabetes self-management education, RRR—relative risk ratio, CI–confidence interval, n–number.
(DOCX)

S6 Table Absolute and weighted relative frequencies of primary reason for not participating in DSME, stratified by socio-demographic and disease-related characteristics and beliefs about diabetes. * The category “not employed” includes students and homemakers as well as retired or disabled respondents; Abbreviations: DMP–Disease-Management-Programme; DSME–structured diabetes self-management education; IPQ-R–Revised Illness Perception Questionnaire-subscale for control belief.
(DOCX)

S7 Table Sensitivity analysis for weighted multinomial logistic regression of primary reason for not participating in DSME on socio-demographic and disease-related characteristics, beliefs and information about diabetes (n = 1396; multiple imputation by chained equations*; only final model). * The proportion of missing information per variable ranged from 0% to 5.7%. For 13.5% of respondents, at least one value was imputed. Abbreviations: DMP–Disease-Management-Programme; DSME–structured diabetes self-management education, RRR–Relative risk ratio.
(DOCX)

S8 Table Perceived benefit of DSME among DSME-participants, relative frequencies of “somewhat/very helpful” by socio-demographic and disease-related characteristics, beliefs and information about diabetes. * The category “not employed” includes students and homemakers as well as retired or disabled respondents; Abbreviations: DMP–Disease-Management-Programme; DSME–structured diabetes self-management education; IPQ-R–Revised Illness Perception Questionnaire-subscale for control belief, CI–confidence interval, n–number.
(DOCX)

S9 Table Weighted logistic regression of perceived benefit of DSME (“somewhat / very helpful” vs. “not at all / rather less helpful”) on socio-demographic and disease-related characteristics, beliefs and information about diabetes (complete case analysis for n = 926). * The category “not employed” includes students and homemakers as well as retired or disabled respondents. Abbreviations: DMP–Disease-Management-Programme; DSME–structured diabetes self-management education; IPQ-R–Revised Illness Perception Questionnaire-subscale for control belief.
(DOCX)

S10 Table Sensitivity analysis for weighted logistic regression of perceived benefit of DSME (“somewhat / very helpful” vs. “not at all / rather less helpful”) on socio-demographic and disease-related characteristics, beliefs and information about diabetes (n = 1002; multiple imputation by chained equations*; only final model). * The proportion of missing information per variable ranged from 0.1% to 1.1%. For 2.1% of respondents, at least one value was imputed. Abbreviations: DMP–Disease-Management-Programme; DSME–structured diabetes self-management education; IPQ-R–Revised Illness Perception Questionnaire-subscale for control belief.
(DOCX)

S11 Table Absolute and weighted relative frequencies of DSME-participation, stratified by DSME-format (individual counselling and group training). Not employed” includes students and homemakers as well as retired or disabled respondents; Abbreviations: DMP–Disease-Management-Programme; DSME–structured diabetes self-management education; IPQ-R–Revised Illness Perception Questionnaire-subscale for control belief.
(DOCX)
