# Supplementary material for: Diabetes self-management education programs: Results from a nationwide population-based study on characteristics of participants, rating of programs and reasons for non-participation
Source: PLoS One. 2024 Sep 12;19(9):e0310338. doi: 10.1371/journal.pone.0310338 (PMC11392325; doi:10.1371/journal.pone.0310338)
Supplement: S1 Table — *The category “not employed” includes students and homemakers as well as retired or disabled respondents; Abbreviations: DMP–Disease-Management-Programme; DSME–structured diabetes self-management education; IPQ-R–Revised Illness Perception Questionnaire-subscale for control belief. (DOCX) [file pone.0310338.s001.docx]

# **Supplemental**

**S1 Table. Absolute and weighted relative frequencies of respondents who ever participated in DSME training and never-participants, stratified by socio-demographic characteristics, disease-related characteristics and beliefs about diabetes**

|  | **never-DSME participants** | | | |  | | **DSME  participants** | | | |  | | **Test for difference** | |
| --- | --- | --- | --- | --- | --- | --- | --- | --- | --- | --- | --- | --- | --- | --- |
|  | **n / n_valid_** | | **f** | |  | | **n / n_valid_** | | **f** | |  | | **p** | |
| **Overall (n = 1396)** | 394 | | 100 % | |  | | 1002 | | 100 % | |  | |  | |
| **Socio-demographic characteristics** | | | | | | | | | | | | | | |
| **Age (n = 1396)** |  | |  | |  | |  | |  | |  | | **p < 0.01** | |
| 18 to 64 years | 88 / 394 | | 34.1 % | |  | | 342 / 1002 | | 47.3 % | |  | |  | |
| 65 to 79 years | 192 / 394 | | 43.1 % | |  | | 493 / 1002 | | 41.1 % | |  | |  | |
| over 80 years | 114 / 394 | | 22.9 % | |  | | 167 / 1002 | | 11.6 % | |  | |  | |
|  |  | |  | |  | |  | |  | |  | |  | |
| **Sex (n = 1396)** |  | |  | |  | |  | |  | |  | | p = 0.223 | |
| Male | 192 / 394 | | 46.8 % | |  | | 527 / 1002 | | 51.4 % | |  | |  | |
| Female | 202 / 394 | | 53.2 % | |  | | 475 / 1002 | | 48.6 % | |  | |  | |
|  |  | |  | |  | |  | |  | |  | |  | |
| **Living situation (n = 1394)** |  | |  | |  | |  | |  | |  | | p = 0.411 | |
| Living alone | 187 / 393 | | 47.2 % | |  | | 425 / 1001 | | 44.1 % | |  | |  | |
| Living together with partner | 206 / 393 | | 52.8 % | |  | | 576 / 1001 | | 55.9 % | |  | |  | |
|  |  | |  | |  | |  | |  | |  | |  | |
| **Educational level (n = 1394)** |  | |  | |  | |  | |  | |  | | p = 0.098 | |
| Low | 126 / 393 | | 52.0 % | |  | | 269 / 1001 | | 44.9 % | |  | |  | |
| Middle | 157 / 393 | | 35.5 % | |  | | 437 / 1001 | | 41.2 % | |  | |  | |
| High | 110 / 393 | | 12.5 % | |  | | 295 / 1001 | | 14.0 % | |  | |  | |
|  |  | |  | |  | |  | |  | |  | |  | |
| **Occupational status (n = 1394)** |  | |  | |  | |  | |  | |  | | **p = 0.014** | |
| Not employed * | 338 / 394 | | 80.2 % | |  | | 783 / 1000 | | 70.6 % | |  | |  | |
| Employed | 56 / 394 | | 19.8 % | |  | | 217 / 1000 | | 29.4 % | |  | |  | |
|  |  | |  | |  | |  | |  | |  | |  | |
| **Residency (n = 1396)** |  | |  | |  | |  | |  | |  | | p = 0.054 | |
| West Germany | 226 / 394 | | 62.6 % | |  | | 659 / 1002 | | 69.2 % | |  | |  | |
| East Germany | 168 / 394 | | 37.4 % | |  | | 343 / 1002 | | 30.8 % | |  | |  | |
| **Disease-related characteristics** | | | | | | | | | | | | | | |
| **Type of Diabetes (n = 1316)** |  | |  | |  | |  | |  | |  | | **p < 0.01** | |
| Type 1 diabetes | 16 / 363 | | 5.3 % | |  | | 151 / 953 | | 81.5 % | |  | |  | |
| Type 2 diabetes | 347 / 363 | | 94.7 % | |  | | 802 / 953 | | 18.5 % | |  | |  | |
|  |  | |  | |  | |  | |  | |  | |  | |
| **Time since diagnosis (n = 1388)** |  | |  | |  | |  | |  | |  | | **p < 0.01** | |
| 2 years or less | 43 / 388 | | 13.5 % | |  | | 43 / 1000 | | 4.8 % | |  | |  | |
| > 2 years to 5 years | 71 / 388 | | 19.8 % | |  | | 114 / 1000 | | 12.6 % | |  | |  | |
| More than 5 years | 274 / 388 | | 66.7 % | |  | | 843 / 1000 | | 82.5 % | |  | |  | |
|  |  | |  | |  | |  | |  | |  | |  | |
| **Non-insulin medication (n = 1396)** |  | |  | |  | |  | |  | |  | | **p = 0.013** | |
| Currently not administered | 108 / 394 | | 30.1 % | |  | | 391 / 1002 | | 39.6 % | |  | |  | |
| Current therapy | 391 / 394 | | 69.9 % | |  | | 611 / 1002 | | 60.4 % | |  | |  | |
|  |  | |  | |  | |  | |  | |  | |  | |
| **Insulin (n = 1395)** |  | |  | |  | |  | |  | |  | | **p < 0.01** | |
| Currently not administered | 287 / 394 | | 66.3 % | |  | | 443 / 1001 | | 43.6 % | |  | |  | |
| Current therapy | 107 / 394 | | 33.7 % | |  | | 558 / 1001 | | 56.4 % | |  | |  | |
|  |  | |  | |  | |  | |  | |  | |  | |
| **Lifestyle therapy (n = 1396)** |  | |  | |  | |  | |  | |  | | p = 0.107 | |
| Currently not administered | 131 / 394 | | 32.7 % | |  | | 254 / 1002 | | 27.4 % | |  | |  | |
| Physical activity and/or dietary therapy | 263 / 394 | | 67.3 % | |  | | 748 / 1002 | | 72.6 % | |  | |  | |
| **Beliefs and information about diabetes** | | | | | | | | |  | |  | |  | |
| **Low perceived risk of diabetes complications (n = 1232)** |  | |  | |  | |  | |  | |  | | p = 0.286 | |
| (Fully / rather) agreement | 199 / 339 | | 56.2 % | |  | | 499 / 893 | | 51.7 % | |  | |  | |
| (Fully / rather) disagreement | 140 / 339 | | 43.8 % | |  | | 394 / 893 | | 48.3 % | |  | |  | |
|  |  | |  | |  | |  | |  | |  | |  | |
| **Personal control subscale (IPQ-R) (n = 1315)** |  | |  | |  | |  | |  | |  | | **p = 0.034** | |
| High (above median of 16) | 131 / 367 | | 34.0 % | |  | | 378 / 948 | | 41.9 % | |  | |  | |
| Low (equal/below median of 16) | 236 / 367 | | 66.0 % | |  | | 570 / 948 | | 58.1 % | |  | |  | |
|  |  | |  | |  | |  | |  | |  | |  | |
| **“I suppose I will have diabetes for the rest of my life” (n = 1386)** |  | |  | |  | |  | |  | |  | | **p < 0.01** | |
| (Fully / rather) agreement | 345 / 390 | | 85.1 % | |  | | 941 / 996 | | 93.2 % | |  | |  | |
| Does not agree (at all) / undecided | 45 / 390 | | 14.9 % | |  | | 55 / 996 | | 6.8 % | |  | |  | |
| **diabetes is a severe disease (n = 1386)** | |  | |  | |  | |  | |  | |  | | **p = 0.019** |
| (Very) severe disease | | 180 / 391 | | 48.3 % | |  | | 552 / 993 | | 57.2 % | |  | |  |
| Not / somewhat severe / no opinion | | 211 / 391 | | 51.7 % | |  | | 443 / 993 | | 42.8 % | |  | |  |
| **Treatment team encouraged to attend any group or training (n = 1384)** | |  | |  | |  | |  | |  | |  | | **p < 0.01** |
| Rarely to always | | 91 / 391 | | 28.3 % | |  | | 606 / 697 | | 66.0 % | |  | |  |
| Never | | 300 / 391 | | 71.7 % | |  | | 387 / 687 | | 34.0 % | |  | |  |
|  | |  | |  | |  | |  | |  | |  | |  |
| **“Are you familiar with DMP?” (n = 1391)** | |  | |  | |  | |  | |  | |  | | **p < 0.01** |
| Yes | | 147 / 394 | | 37.9 % | |  | | 550 / 997 | | 54.3 % | |  | |  |
| No | | 247 / 394 | | 62.1 % | |  | | 447 / 997 | | 45.7 % | |  | |  |
|  | |  | |  | |  | |  | |  | |  | |  |

* The category “not employed” includes students and homemakers as well as retired or disabled respondents;

Abbreviations: DMP – Disease-Management-Programme; DSME – structured diabetes self-management education; IPQ-R – Revised Illness Perception Questionnaire-subscale for control belief
