# Supplementary material for: Diabetes self-management education programs: Results from a nationwide population-based study on characteristics of participants, rating of programs and reasons for non-participation
Source: PLoS One. 2024 Sep 12;19(9):e0310338. doi: 10.1371/journal.pone.0310338 (PMC11392325; doi:10.1371/journal.pone.0310338)
Supplement: S2 Table — *The category “not employed” includes students and homemakers as well as retired or disabled respondents; Abbreviations: DMP–Disease-Management-Programme; DSME–structured diabetes self-management education; IPQ-R–Revised Illness Perception Questionnaire-subscale for control belief. (DOCX) [file pone.0310338.s002.docx]

**S2 Table. Weighted logistic regression of DSME-participation on socio-demographic and disease-related characteristics, beliefs and information about diabetes (complete case analysis for n = 1210)**

|  | **initial model** | | | | **final model** | | | |
| --- | --- | --- | --- | --- | --- | --- | --- | --- |
|  | **OR** | **95 % C.I.** | | **p** | **OR** | **95 % C.I.** | | **p** |
| **Socio-demographic characteristics** |  |  |  |  |  |  |  |  |
| 65 to 79 years (vs. 18 to 64 years) | 0.96 | [0.55; | 1.67] | 0.879 |  |  |  |  |
| Over 80 years (vs. 18 to 64 years) | 0.73 | [0.38; | 1.42] | 0.355 |  |  |  |  |
| Female (vs. male) | 1.17 | [0.82; | 1.67] | 0.399 |  |  |  |  |
| Middle educational level (vs. low educational level) | **1.67** | **[1.07;** | **2.63]** | **0.025** | **1.82** | **[1.21;** | **2.73]** | **0.004** |
| High educational level (vs. low educational level) | **1.97** | **[1.23;** | **3.17]** | **0.005** | **2.04** | **[1.30;** | **3.21]** | **0.002** |
| Not employed (vs. employed) * | 0.88 | [0.45; | 1.71] | 0.697 |  |  |  |  |
| East Germany (vs. West Germany) | **0.59** | **[0.40;** | **0.86]** | **0.007** | **0.57** | **[0.39;** | **0.83]** | **0.003** |
| **Disease-related characteristics** |  |  |  |  |  |  |  |  |
| Type 1 diabetes (Type 2 diabetes) | 2.11 | [1.00; | 4.45] | 0.051 | **2.46** | **[1.24;** | **4.90]** | **0.010** |
| 2 years or less since diagnosis (vs. more than 5 years) | **0.45** | **[0.22;** | **0.93]** | **0.031** | 0.51 | [0.25; | 1.04] | 0.063 |
| > 2 years to 5 years since diagnosis (vs. more than 5 years) | **0.49** | **[0.29;** | **0.82]** | **0.007** | **0.52** | **[0.31;** | **0.88]** | **0.014** |
| Non-Insulin medication (vs. currently not administered) | 0.89 | [0.54; | 1.45] | 0.628 |  |  |  |  |
| Insulin (vs. currently not administered) | **1.91** | **[1.22;** | **3.00]** | **0.005** | **1.96** | **[1.33;** | **2.90]** | **0.001** |
| Lifestyle therapy (vs. currently not administered) | 1.09 | [0.72; | 1.63] | 0.692 |  |  |  |  |
| **Beliefs and information about diabetes** |  |  |  |  |  |  |  |  |
| Low personal control belief (IPQ-R≤16 vs. IPQ-R>16) | 0.82 | [0.55; | 1.22] | 0.335 |  |  |  |  |
| No agreement /undecided that diabetes will be present for the rest of life  (vs. agreement) | **0.27** | **[0.13;** | **0.56]** | **<0.001** | **0.30** | **[0.15;** | **0.62]** | **0.001** |
| Diabetes not regarded as severe disease / no opinion (vs. severe disease) | 0.91 | [0.63; | 1.31] | 0.607 |  |  |  |  |
| Never being encouraged to attend any group or training (vs. rarely to always) | **0.19** | **[0.13;** | **0.29]** | **<0.001** | **0.19** | **[0.13;** | **0.27]** | **<0.001** |
| Not familiar with DMP (vs. familiar with DMP) | **0.69** | **[0.49;** | **0.98]** | **0.040** | **0.67** | **[0.47;** | **0.96]** | **0.029** |
| n | 1210 |  |  |  | 1210 |  |  |  |
| c statistic | 0.796 |  |  |  | 0.790 |  |  |  |

* The category “not employed” includes students and homemakers as well as retired or disabled respondents; Abbreviations: DMP – Disease-Management-Programme; DSME – structured diabetes self-management education; IPQ-R – Revised Illness Perception Questionnaire-subscale for control belief
