# Supplementary material for: Diabetes self-management education programs: Results from a nationwide population-based study on characteristics of participants, rating of programs and reasons for non-participation
Source: PLoS One. 2024 Sep 12;19(9):e0310338. doi: 10.1371/journal.pone.0310338 (PMC11392325; doi:10.1371/journal.pone.0310338)
Supplement: S3 Table — * The proportion of missing information per variable ranged from 0% to 5.7%. For 13.3% of respondents, at least one value was imputed. Abbreviations: DMP–Disease-Management-Programme; DSME–structured diabetes self-management education, OR–Odds ratio. (DOCX) [file pone.0310338.s003.docx]

**S3 Table: Sensitivity analyses for weighted logistic regression of DSME-participation on socio-demographic and disease-related characteristics, beliefs and information about diabetes (n = 1396; multiple imputation by chained equations*)**

|  | **final model** | | | |
| --- | --- | --- | --- | --- |
|  | **OR** | **95 % CI** | | **p** |
| **Socio-demographic characteristics** |  |  |  |  |
| Middle educational level (vs. low educational level) | **1.60** | **[1.10;** | **2.33]** | **0.014** |
| High educational level (vs. low educational level) | **1.69** | **[1.12;** | **2.54]** | **0.012** |
| East Germany (vs. West Germany) | **0.61** | **[0.43;** | **0.85]** | **0.004** |
| **Disease-related factors** |  |  |  |  |
| Type 1 diabetes ( vs.type 2 diabetes) | **2.49** | **[1.27;** | **4.88]** | **0.008** |
| 2 years or less since diagnosis (vs. more than 5 years) | **0.41** | **[0.22;** | **0.75]** | **0.004** |
| > 2 years to 5 years since diagnosis (vs. more than 5 years) | **0.55** | **[0.34;** | **0.91]** | **0.019** |
| Insulin (vs. currently not administered) | **1.91** | **[1.31;** | **2.80]** | **0.001** |
| **Beliefs and information about diabetes** |  |  |  |  |
| No agreement /undecided that diabetes will be present for the rest of life (vs. agreement) | **0.46** | **[0.22;** | **0.97]** | **0.041** |
| Not familiar with DMP (vs. familiar with DMP) | **0.20** | **[0.14;** | **0.28]** | **0.000** |
| Never being encouraged to attend any group or training (vs. rarely to always) | **0.63** | **[0.45;** | **0.89]** | **0.008** |
| n | 1396 |  |  |  |
| c statistic | 0.786 |  |  |  |
|  |  |  |  |  |

* The proportion of missing information per variable ranged from 0 % to 5.7 %. For 13.3 % of respondents, at least one value was imputed.

Abbreviations: DMP – Disease-Management-Programme; DSME – structured diabetes self-management education, OR – Odds ratio
