# Supplementary material for: Diabetes self-management education programs: Results from a nationwide population-based study on characteristics of participants, rating of programs and reasons for non-participation
Source: PLoS One. 2024 Sep 12;19(9):e0310338. doi: 10.1371/journal.pone.0310338 (PMC11392325; doi:10.1371/journal.pone.0310338)
Supplement: S4 Table — * The category “not employed” includes students and homemakers as well as retired or disabled respondents; Abbreviations: DMP–Disease-Management-Programme; DSME–structured diabetes self-management education; IPQ-R–Revised Illness Perception Questionnaire-subscale for control belief. (DOCX) [file pone.0310338.s004.docx]

**S4 Table: Absolute and weighted relative frequencies of respondents who ever participated in DSME training and never-participants, stratified by socio-demographic characteristics, disease-related characteristics and beliefs about diabetes**

|  | **never-DSME participants** | |  | **DSME  participants** | |  | **Test for difference** |
| --- | --- | --- | --- | --- | --- | --- | --- |
|  | **n / n_valid_** | **f** |  | **n / n_valid_** | **f** |  | **p** |
| **Overall (n = 1396)** | 394 | 100 % |  | 1002 | 100 % |  |  |
| **Socio-demographic characteristics** | | | | | | | |
| **Age (n = 1396)** |  |  |  |  |  |  | **p < 0.01** |
| 18 to 64 years | 88 / 394 | 34.1 % |  | 342 / 1002 | 47.3 % |  |  |
| 65 to 79 years | 192 / 394 | 43.1 % |  | 493 / 1002 | 41.1 % |  |  |
| over 80 years | 114 / 394 | 22.9 % |  | 167 / 1002 | 11.6 % |  |  |
|  |  |  |  |  |  |  |  |
| **Sex (n = 1396)** |  |  |  |  |  |  | p = 0.223 |
| Male | 192 / 394 | 46.8 % |  | 527 / 1002 | 51.4 % |  |  |
| Female | 202 / 394 | 53.2 % |  | 475 / 1002 | 48.6 % |  |  |
|  |  |  |  |  |  |  |  |
| **Living situation (n = 1394)** |  |  |  |  |  |  | p = 0.411 |
| Living alone | 187 / 393 | 47.2 % |  | 425 / 1001 | 44.1 % |  |  |
| Living together with partner | 206 / 393 | 52.8 % |  | 576 / 1001 | 55.9 % |  |  |
|  |  |  |  |  |  |  |  |
| **Educational level (n = 1394)** |  |  |  |  |  |  | p = 0.098 |
| Low | 126 / 393 | 52.0 % |  | 269 / 1001 | 44.9 % |  |  |
| Middle | 157 / 393 | 35.5 % |  | 437 / 1001 | 41.2 % |  |  |
| High | 110 / 393 | 12.5 % |  | 295 / 1001 | 14.0 % |  |  |
|  |  |  |  |  |  |  |  |
| **Occupational status (n = 1394)** |  |  |  |  |  |  | **p = 0.014** |
| Not employed * | 338 / 394 | 80.2 % |  | 783 / 1000 | 70.6 % |  |  |
| Employed | 56 / 394 | 19.8 % |  | 217 / 1000 | 29.4 % |  |  |
|  |  |  |  |  |  |  |  |
| **Residency (n = 1396)** |  |  |  |  |  |  | p = 0.054 |
| West Germany | 226 / 394 | 62.6 % |  | 659 / 1002 | 69.2 % |  |  |
| East Germany | 168 / 394 | 37.4 % |  | 343 / 1002 | 30.8 % |  |  |
| **Disease-related characteristics** | | | | | | | |
| **Type of Diabetes (n = 1316)** |  |  |  |  |  |  | **p < 0.01** |
| Type 1 diabetes | 16 / 363 | 5.3 % |  | 151 / 953 | 81.5 % |  |  |
| Type 2 diabetes | 347 / 363 | 94.7 % |  | 802 / 953 | 18.5 % |  |  |
|  |  |  |  |  |  |  |  |
| **Time since diagnosis (n = 1388)** |  |  |  |  |  |  | **p < 0.01** |
| 2 years or less | 43 / 388 | 13.5 % |  | 43 / 1000 | 4.8 % |  |  |
| > 2 years to 5 years | 71 / 388 | 19.8 % |  | 114 / 1000 | 12.6 % |  |  |
| More than 5 years | 274 / 388 | 66.7 % |  | 843 / 1000 | 82.5 % |  |  |
|  |  |  |  |  |  |  |  |
| **Non-insulin medication (n = 1396)** |  |  |  |  |  |  | **p = 0.013** |
| Currently not administered | 108 / 394 | 30.1 % |  | 391 / 1002 | 39.6 % |  |  |
| Current therapy | 391 / 394 | 69.9 % |  | 611 / 1002 | 60.4 % |  |  |
|  |  |  |  |  |  |  |  |
| **Insulin (n = 1395)** |  |  |  |  |  |  | **p < 0.01** |
| Currently not administered | 287 / 394 | 66.3 % |  | 443 / 1001 | 43.6 % |  |  |
| Current therapy | 107 / 394 | 33.7 % |  | 558 / 1001 | 56.4 % |  |  |
|  |  |  |  |  |  |  |  |
| **Lifestyle therapy (n = 1396)** |  |  |  |  |  |  | p = 0.107 |
| Currently not administered | 131 / 394 | 32.7 % |  | 254 / 1002 | 27.4 % |  |  |
| Physical activity and/or dietary therapy | 263 / 394 | 67.3 % |  | 748 / 1002 | 72.6 % |  |  |
| **Beliefs and information about diabetes** | | | | |  |  |  |
| **Low perceived risk of diabetes complications (n = 1232)** |  |  |  |  |  |  | p = 0.286 |
| (Fully / rather) agreement | 199 / 339 | 56.2 % |  | 499 / 893 | 51.7 % |  |  |
| (Fully / rather) disagreement | 140 / 339 | 43.8 % |  | 394 / 893 | 48.3 % |  |  |
|  |  |  |  |  |  |  |  |
| **Personal control subscale (IPQ-R) (n = 1315)** |  |  |  |  |  |  | **p = 0.034** |
| High (above median of 16) | 131 / 367 | 34.0 % |  | 378 / 948 | 41.9 % |  |  |
| Low (equal/below median of 16) | 236 / 367 | 66.0 % |  | 570 / 948 | 58.1 % |  |  |
|  |  |  |  |  |  |  |  |
| **“I suppose I will have diabetes for the rest of my life” (n = 1386)** |  |  |  |  |  |  | **p < 0.01** |
| (Fully / rather) agreement | 345 / 390 | 85.1 % |  | 941 / 996 | 93.2 % |  |  |
| Does not agree (at all) / undecided | 45 / 390 | 14.9 % |  | 55 / 996 | 6.8 % |  |  |
| **diabetes is a severe disease (n = 1386)** |  |  |  |  |  |  | p = 0.019 |
| (Very) severe disease | 180 / 391 | 48.3 % |  | 552 / 993 | 57.2 % |  |  |
| Not / somewhat severe / no opinion | 211 / 391 | 51.7 % |  | 443 / 993 | 42.8 % |  |  |
| **Treatment team encouraged to attend any group or training (n = 1384)** |  |  |  |  |  |  | **p < 0.01** |
| Rarely to always | 91 / 391 | 28.3 % |  | 606 / 697 | 66.0 % |  |  |
| Never | 300 / 391 | 71.7 % |  | 387 / 687 | 34.0 % |  |  |
|  |  |  |  |  |  |  |  |
| **“Are you familiar with DMP?” (n = 1391)** |  |  |  |  |  |  | **p < 0.01** |
| Yes | 147 / 394 | 37.9 % |  | 550 / 997 | 54.3 % |  |  |
| No | 247 / 394 | 62.1 % |  | 447 / 997 | 45.7 % |  |  |
|  |  |  |  |  |  |  |  |

* The category “not employed” includes students and homemakers as well as retired or disabled respondents;

Abbreviations: DMP – Disease-Management-Programme; DSME – structured diabetes self-management education; IPQ-R – Revised Illness Perception Questionnaire-subscale for control belief
