# Supplementary material for: Diabetes self-management education programs: Results from a nationwide population-based study on characteristics of participants, rating of programs and reasons for non-participation
Source: PLoS One. 2024 Sep 12;19(9):e0310338. doi: 10.1371/journal.pone.0310338 (PMC11392325; doi:10.1371/journal.pone.0310338)
Supplement: S5 Table — Abbreviations: DMP–Disease-Management-Programme; DSME–structured diabetes self-management education, RRR—relative risk ratio, CI–confidence interval, n–number. (DOCX) [file pone.0310338.s005.docx]

**S5 Table: Weighted multinomial logistic regression of main reason for not participating in DSME on socio-demographic and disease-related characteristics, beliefs and information about diabetes (complete case analysis for n = 1208; only final model)**

|  | **Lack of information or recommendation  (Ref: DSME  participants)** | | | | **Further reasons for not participating (Ref: DSME  participants)** | | | |
| --- | --- | --- | --- | --- | --- | --- | --- | --- |
|  | **RRR** | **95 % C.I.** | | **p** | **RRR** | **95 % C.I.** | | **p** |
| **Socio-demographic characteristics** |  |  |  |  |  |  |  |  |
| 65 to 79 years (vs. 18 to 64 years) | 1.20 | [0.69; | 2.08] | 0.510 | 1.36 | [0.76; | 2.43] | 0.294 |
| Over 80 years (vs. 18 to 64 years) | **2.10** | **[1.09;** | **4.03]** | **0.026** | 1.63 | [0.81; | 3.25] | 0.168 |
| East Germany (vs. West Germany) | **1.64** | **[1.05;** | **2.55]** | **0.029** | 1.42 | [0.88; | 2.30] | 0.155 |
| **Disease-related characteristics** |  |  |  |  |  |  |  |  |
| Type 1 diabetes (vs. type 2 diabetes) | **0.24** | **[0.08;** | **0.73]** | **0.012** | 0.44 | [0.18; | 1.05] | 0.065 |
| 2 years or less since diagnosis (vs. more than 5 years) | 2.15 | [0.86; | 5.37] | 0.100 | 1.69 | [0.73; | 3.92] | 0.224 |
| > 2 years to 5 years since diagnosis (vs. more than 5 years) | **2.36** | **[1.25;** | **4.45]** | **0.008** | 1.50 | [0.81; | 2.77] | 0.195 |
| Insulin (vs. currently not administered) | 0.66 | [0.40; | 1.09] | 0.102 | **0.39** | **[0.23;** | **0.66]** | **<0.001** |
| **Beliefs and information about diabetes** |  |  |  |  |  |  |  |  |
| No agreement /undecided that diabetes will be present for the rest of life  (vs. agreement) | **5.67** | **[2.44;** | **13.17]** | **<0.001** | 2.05 | [0.95; | 4.42] | 0.066 |
| Never encouraged to attend any group or training (vs. rarely to always) | **3.91** | **[2.46;** | **6.21]** | **<0.001** | **5.18** | **[3.18;** | **8.44]** | **<0.001** |
| Not familiar with DMP (vs. familiar with DMP) | **2.89** | **[1.79;** | **4.69]** | **<0.001** | 0.91 | [0.59; | 1.42] | 0.686 |

Abbreviations: DMP – Disease-Management-Programme; DSME – structured diabetes self-management education, RRR - relative risk ratio, CI – confidence interval, n - number
