# Supplementary material for: Diabetes self-management education programs: Results from a nationwide population-based study on characteristics of participants, rating of programs and reasons for non-participation
Source: PLoS One. 2024 Sep 12;19(9):e0310338. doi: 10.1371/journal.pone.0310338 (PMC11392325; doi:10.1371/journal.pone.0310338)
Supplement: S6 Table — * The category “not employed” includes students and homemakers as well as retired or disabled respondents; Abbreviations: DMP–Disease-Management-Programme; DSME–structured diabetes self-management education; IPQ-R–Revised Illness Perception Questionnaire-subscale for control belief. (DOCX) [file pone.0310338.s006.docx]

**S6 Table: Absolute and weighted relative frequencies of primary reason for not participating in DSME, stratified by socio-demographic and disease-related characteristics and beliefs about diabetes**

|  | **DSME  participants** | |  | **Lack of information or recommendation** | |  | **Further reasons for not participating in DSME** | |  | **Test for difference** |
| --- | --- | --- | --- | --- | --- | --- | --- | --- | --- | --- |
|  | **n / n_valid_** | **f** |  | **n / n_valid_** | **f** |  | **n / n_valid_** | **f** |  | **p** |
| **Overall (n = 1391)** | 1002 / 1391 | 73.4 % |  | 193 / 1391 | 12.9 % |  | 196 / 1391 | 13.7 % |  |  |
| **Age (n = 1391)** |  |  |  |  |  |  |  |  |  | **p < 0.01** |
| 18 to 64 years | 342 / 428 | 79.7 % |  | 41 / 428 | 10.1 % |  | 45 / 428 | 10.2 % |  |  |
| 65 to 79 years | 493 / 683 | 72.3 % |  | 92 / 683 | 13.1 % |  | 98 / 683 | 14.6 % |  |  |
| over 80 years | 167 / 280 | 58.0 % |  | 60 / 280 | 21.0 % |  | 53 / 280 | 21.0 % |  |  |
| **Sex (n = 1391)** |  |  |  |  |  |  |  |  |  | **p = 0.015** |
| Male | 527 / 717 | 75.2 % |  | 84 / 717 | 9.9 % |  | 106 / 717 | 14.9 % |  |  |
| Female | 475 / 674 | 71.6 % |  | 109 / 674 | 16.0 % |  | 90 / 674 | 12.4 % |  |  |
| **Living situation (n = 1389)** |  |  |  |  |  |  |  |  |  | p = 0.486 |
| Living alone | 425 / 609 | 72.0 % |  | 90 / 609 | 14.3 % |  | 94 / 609 | 13.8 % |  |  |
| Living together with partner | 576 / 780 | 74.7 % |  | 102 / 780 | 11.6 % |  | 102 / 780 | 13.6 % |  |  |
| **Educational level (n = 1389)** |  |  |  |  |  |  |  |  |  | p = 0.379 |
| Low | 269 / 392 | 70.8 % |  | 58 / 392 | 13.8 % |  | 65 / 392 | 15.5 % |  |  |
| Middle | 437 / 593 | 75.9 % |  | 82 / 593 | 12.2 % |  | 74 / 593 | 11.9 % |  |  |
| High | 295 / 404 | 75.3 % |  | 53 / 404 | 12.3 % |  | 56 / 404 | 12.4 % |  |  |
| **Occupational status (n = 1389)** |  |  |  |  |  |  |  |  |  | p = 0.072 |
| Not employed * | 783 / 1116 | 70.9 % |  | 172 / 1116 | 14.4 % |  | 161 / 1116 | 14.6 % |  |  |
| Employed | 217 / 273 | 80.0 % |  | 21 / 273 | 8.9 % |  | 35 / 273 | 11.1 % |  |  |
| **Residency (n = 1391)** |  |  |  |  |  |  |  |  |  | p = 0.069 |
| West Germany | 659 / 882 | 75.5 % |  | 103 / 882 | 11.3 % |  | 120 / 882 | 13.2 % |  |  |
| East Germany | 343 / 509 | 69.1 % |  | 90 / 509 | 16.3 % |  | 76 / 509 | 14.6 % |  |  |
| **Type of Diabetes (n = 1312)** |  |  |  |  |  |  |  |  |  | **p < 0.01** |
| Type 1 diabetes | 151 / 166 | 92.3 % |  | 4 / 166 | 2.5 % |  | 11 / 166 | 5.2 % |  |  |
| Type 2 diabetes | 802 / 1146 | 70.7 % |  | 173 / 1146 | 14.2 % |  | 171 / 1146 | 15.1 % |  |  |
| **Time since diagnosis (n = 1383)** |  |  |  |  |  |  |  |  |  | **p < 0.01** |
| 2 years or less | 43 / 85 | 50.5 % |  | 21 / 85 | 26.0 % |  | 21 / 85 | 23.4 % |  |  |
| > 2 years to 5 years | 114 / 184 | 63.7 % |  | 35 / 184 | 20.6 % |  | 35 / 184 | 15.7 % |  |  |
| More than 5 years | 843 / 1114 | 77.5 % |  | 135 / 1114 | 10.3 % |  | 136 / 1114 | 12.2 % |  |  |
| **Non-insulin medication (n = 1391)** |  |  |  |  |  |  |  |  |  | p = 0.019 |
| Currently not administered | 391 / 496 | 79.1 % |  | 57 / 496 | 10.4 % |  | 48 / 496 | 10.5 % |  |  |
| Current therapy | 611 / 895 | 70.1 % |  | 136 / 895 | 14.4 % |  | 148 / 895 | 15.5 % |  |  |
| **Insulin (n = 1390)** |  |  |  |  |  |  |  |  |  | **p < 0.01** |
| Currently not administered | 443 / 728 | 64.0 % |  | 132 / 728 | 15.8 % |  | 153 / 728 | 20.1 % |  |  |
| Current therapy | 558 / 662 | 82.7 % |  | 61 / 662 | 10.1 % |  | 43 / 662 | 7.2 % |  |  |
| **Lifestyle therapy (n = 1391)** |  |  |  |  |  |  |  |  |  | p = 0.180 |
| Currently not administered | 254 / 384 | 69.7 % |  | 66 / 384 | 15.8 % |  | 64 / 384 | 14.5 % |  |  |
| Physical activity and/or dietary therapy | 748 / 1007 | 74.9 % |  | 127 / 1007 | 11.8 % |  | 132 / 1007 | 13.3 % |  |  |
| **Low perceived risk of diabetes complications (n = 1228)** |  |  |  |  |  |  |  |  |  | p = 0.410 |
| (Fully / rather) agreement | 499 / 697 | 71.9 % |  | 100 / 697 | 13.6 % |  | 98 / 697 | 14.5 % |  |  |
| (Fully / rather) disagreement | 394 / 531 | 76.3 % |  | 63 / 531 | 11.6 % |  | 74 / 531 | 12.1 % |  |  |
| **Personal control subscale (IPQ-R) (n = 1310)** |  |  |  |  |  |  |  |  |  | p = 0.083 |
| High (above median of 16) | 378 / 508 | 77.5 % |  | 62 / 508 | 10.7 % |  | 68 / 508 | 11.8 % |  |  |
| Low (equal/below median of 16) | 570 / 802 | 70.9 % |  | 116 / 802 | 14.3 % |  | 116 / 802 | 14.8 % |  |  |
| **“I suppose I will have diabetes for the rest of my life” (n = 1381)** |  |  |  |  |  |  |  |  |  | **p < 0.01** |
| (Fully / rather) agreement | 941 / 1283 | 75.2 % |  | 167 / 1283 | 11.2 % |  | 175 / 1283 | 13.6 % |  |  |
| Does not agree (at all) / undecided | 55 / 98 | 55.9 % |  | 24 / 98 | 30.0 % |  | 19 / 98 | 14.1 % |  |  |
| **“I consider diabetes to be a serious disease” (n = 1381)** |  |  |  |  |  |  |  |  |  | p = 0.093 |
| (Very) severe disease | 552 / 732 | 76.2 % |  | 101 / 732 | 12.2 % |  | 79 / 732 | 11.7 % |  |  |
| Not / somewhat severe / no opinion | 443 / 649 | 69.9 % |  | 92 / 649 | 14.0 % |  | 114 / 649 | 16.1 % |  |  |
| **Treatment team encouraged to attend any group or training (n = 1379)** |  |  |  |  |  |  |  |  |  | **p < 0.01** |
| Rarely to always | 606 / 697 | 86.3 % |  | 42 / 697 | 7.1 % |  | 49 / 697 | 6.6 % |  |  |
| Never | 387 / 682 | 56.9 % |  | 149 / 682 | 20.3 % |  | 146 / 682 | 22.8 % |  |  |
| **“Are you familiar with DMP?” (n = 1386)** |  |  |  |  |  |  |  |  |  | **p < 0.01** |
| Yes | 550 / 695 | 80.1 % |  | 51 / 695 | 6.4 % |  | 94 / 695 | 13.6 % |  |  |
| No | 447 / 691 | 66.7 % |  | 142 / 691 | 19.5 % |  | 102 / 691 | 13.9 % |  |  |
|  |  |  |  |  |  |  |  |  |  |  |

* The category “not employed” includes students and homemakers as well as retired or disabled respondents; Abbreviations: DMP – Disease-Management-Programme; DSME – structured diabetes self-management education; IPQ-R – Revised Illness Perception Questionnaire-subscale for control belief
