# Supplementary material for: Diabetes self-management education programs: Results from a nationwide population-based study on characteristics of participants, rating of programs and reasons for non-participation
Source: PLoS One. 2024 Sep 12;19(9):e0310338. doi: 10.1371/journal.pone.0310338 (PMC11392325; doi:10.1371/journal.pone.0310338)
Supplement: S7 Table — * The proportion of missing information per variable ranged from 0% to 5.7%. For 13.5% of respondents, at least one value was imputed. Abbreviations: DMP–Disease-Management-Programme; DSME–structured diabetes self-management education, RRR–Relative risk ratio. (DOCX) [file pone.0310338.s007.docx]

**S7 Table. Sensitivity analysis for weighted multinomial logistic regression of primary reason for not participating in DSME on socio-demographic and disease-related characteristics, beliefs and information about diabetes (n = 1396; multiple imputation by chained equations*; only final model)**

|  | **Lack of information or recommendation  (Ref: DSME  participants)** | | | |  | **Further reasons for not participating (Ref: DSME  participants)** | | | |
| --- | --- | --- | --- | --- | --- | --- | --- | --- | --- |
|  | **RRR** | **95 % CI** | | **p** |  | **RRR** | **95 % CI** | | **p** |
| **Socio-demographic characteristics** |  |  |  |  |  |  |  |  |  |
| 65 to 79 years (vs. 18 to 64 years) | 1.40 | [0.83; | 2.36] | 0.203 |  | 1.46 | [0.83; | 2.55] | 0.188 |
| Over 80 years (vs. 18 to 64 years) | **2.12** | **[1.16;** | **3.88]** | **0.014** |  | **2.10** | **[1.10;** | **3.99]** | **0.024** |
| East Germany (vs. West Germany) | **1.72** | **[1.16;** | **2.56]** | **0.008** |  | 1.25 | [0.80; | 1.97] | 0.330 |
| **Disease-related characteristics** |  |  |  |  |  |  |  |  |  |
| Type 1 diabetes (Type 2 diabetes) | **0.23** | **[0.07;** | **0.76]** | **0.016** |  | 0.45 | [0.19; | 1.05] | 0.065 |
| 2 years or less since diagnosis (vs. more than 5 years) | **2.91** | **[1.31;** | **6.49]** | **0.009** |  | **2.39** | **[1.14;** | **5.00]** | **0.021** |
| > 2 years to 5 years since diagnosis (vs. more than 5 years) | **2.23** | **[1.22;** | **4.08]** | **0.009** |  | 1.51 | [0.88; | 2.61] | 0.136 |
| Insulin (vs. currently not administered) | 0.72 | [0.43; | 1.19] | 0.199 |  | **0.37** | **[0.22;** | **0.61]** | **<0.001** |
| **Beliefs and information about diabetes** |  |  |  |  |  |  |  |  |  |
| No agreement /undecided that diabetes will be present for the rest of life (vs. agreement) | **3.90** | **[1.74;** | **8.75]** | **0.001** |  | 1.47 | [0.69; | 3.12] | 0.316 |
| Never being encouraged to attend any group or training (vs. rarely to always) | **3.83** | **[2.46;** | **5.95]** | **<0.001** |  | **4.82** | **[3.09;** | **7.53]** | **<0.001** |
| Not familiar with DMP (vs. familiar with DMP) | **2.77** | **[1.75;** | **4.39]** | **<0.001** |  | 0.98 | [0.65; | 1.47] | 0.917 |
| n | 1396 |  |  |  |  |  |  |  |  |

* The proportion of missing information per variable ranged from 0% to 5.7 %. For 13.5 % of respondents, at least one value was imputed. Abbreviations: DMP – Disease-Management-Programme; DSME – structured diabetes self-management education, RRR – Relative risk ratio
