# Supplementary material for: Diabetes self-management education programs: Results from a nationwide population-based study on characteristics of participants, rating of programs and reasons for non-participation
Source: PLoS One. 2024 Sep 12;19(9):e0310338. doi: 10.1371/journal.pone.0310338 (PMC11392325; doi:10.1371/journal.pone.0310338)
Supplement: S8 Table — * The category “not employed” includes students and homemakers as well as retired or disabled respondents; Abbreviations: DMP–Disease-Management-Programme; DSME–structured diabetes self-management education; IPQ-R–Revised Illness Perception Questionnaire-subscale for control belief, CI–confidence interval, n–number. (DOCX) [file pone.0310338.s008.docx]

**Table S8.** **Perceived benefit of DSME among DSME-participants, relative frequencies of “somewhat/very helpful” by socio-demographic and disease-related characteristics, beliefs and information about diabetes**

|  | **“somewhat or very helpful”  / n** | |  | | **weighted relative frequency** | | | | |  | | **Test for difference** | |
| --- | --- | --- | --- | --- | --- | --- | --- | --- | --- | --- | --- | --- | --- |
|  |  | |  | | **f** | | **95 % C.I.** | | |  | | **p** | |
| **Overall (n = 991; missing for n = 11)** | 896 / 991 | |  | | 90.2 % | | [87.6 %; | | 92.3 %] |  | |  | |
| **Socio-demographic characteristics** | | | | | | | | | | | | | |
| **Age (n = 991)** |  | |  | |  | |  | |  |  | | p = 0.306 | |
| 18 to 64 years | 311 / 339 | |  | | 91.7 % | | [87.2 %; | | 94.7 %] |  | |  | |
| 65 to 79 years | 439 / 486 | |  | | 89.6 % | | [85.6 %; | | 92.5 %] |  | |  | |
| over 80 years | 146 / 166 | |  | | 86.3 % | | [78.6 %; | | 91.5 %] |  | |  | |
|  |  | |  | |  | |  | |  |  | |  | |
| **Sex (n = 991)** |  | |  | |  | |  | |  |  | | **p = 0.046** | |
| Male | 481 / 520 | |  | | 92.6 % | | [88.8 %; | | 95.2 %] |  | |  | |
| Female | 415 / 471 | |  | | 87.7 % | | [83.6 %; | | 90.8 %] |  | |  | |
|  |  | |  | |  | |  | |  |  | |  | |
| **Living situation (n = 990)** |  | |  | |  | |  | |  |  | | p = 0.054 | |
| Living alone | 372 / 424 | |  | | 87.6 % | | [82.9 %; | | 91.2 %] |  | |  | |
| Living together with partner | 523 / 566 | |  | | 92.2 % | | [89.1 %; | | 94.5 %] |  | |  | |
|  |  | |  | |  | |  | |  |  | |  | |
| **Educational level (n = 990)** |  | |  | |  | |  | |  |  | | p = 0.054 | |
| Low | 233 / 267 | |  | | 87.5 % | | [82.4 %; | | 91.3 %] |  | |  | |
| Middle | 400 / 433 | |  | | 92.9 % | | [89.7 %; | | 95.1 %] |  | |  | |
| High | 262 / 290 | |  | | 90.8 % | | [86.1 %; | | 94.1 %] |  | |  | |
|  |  | |  | |  | |  | |  |  | |  | |
| **Occupational status (n = 989)** |  | |  | |  | |  | |  |  | | p = 0.071 | |
| Not employed * | 695 / 773 | |  | | 88.8 % | | [85.4 %; | | 91.5 %] |  | |  | |
| Employed | 199 / 216 | |  | | 93.4 % | | [89.0 %; | | 96.1 %] |  | |  | |
|  |  | |  | |  | |  | |  |  | |  | |
| **Residency (n = 991)** |  | |  | |  | |  | |  |  | | p = 0.198 | |
| West Germany | 585 / 652 | |  | | 89.3 % | | [85.8 %; | | 92.0 %] |  | |  | |
| East Germany | 311 / 339 | |  | | 92.3 % | | [88.4 %; | | 94.9 %] |  | |  | |
| **Disease-related characteristics** | | | | | | | | | | | | | |
| **Type of Diabetes (n = 943)** |  | |  | |  | |  | |  |  | | p = 0.628 | |
| Type 1 diabetes | 138 / 151 | |  | | 88.7 % | | [78.1 %; | | 94.5 %] |  | |  | |
| Type 2 diabetes | 716 / 792 | |  | | 90.6 % | | [87.9 %; | | 92.7 %] |  | |  | |
|  |  | |  | |  | |  | |  |  | |  | |
| **Time since diagnosis (n = 989)** |  | |  | |  | |  | |  |  | | p = 0.615 | |
| 2 years or less | 38 / 42 | |  | | 90.3 % | | [74.7 %; | | 96.7 %] |  | |  | |
| > 2 years to 5 years | 106 / 113 | |  | | 93.2 % | | [84.7 %; | | 97.2 %] |  | |  | |
| More than 5 years | 750 / 834 | |  | | 89.7 % | | [86.7 %; | | 92.1 %] |  | |  | |
|  |  | |  | |  | |  | |  |  | |  | |
| **Non-insulin medication (n = 991)** |  | |  | |  | |  | |  |  | | p = 0.181 | |
| Currently not administered | 349 / 388 | |  | | 88.2 % | | [82.9 %; | | 92.0 %] |  | |  | |
| Current therapy | 547 / 603 | |  | | 91.5 % | | [88.6 %; | | 93.7 %] |  | |  | |
|  |  | |  | |  | |  | |  |  | |  | |
| **Insulin (n = 990)** |  | |  | |  | |  | |  |  | | p = 0.832 | |
| Currently not administered | 394 / 439 | |  | | 90.5 % | | [86.8 %; | | 93.2 %] |  | |  | |
| Current therapy | 501 / 551 | |  | | 90.0 % | | [86.0 %; | | 92.9 %] |  | |  | |
|  |  | |  | |  | |  | |  |  | |  | |
| **Lifestyle therapy (n = 991)** |  | |  | |  | |  | |  |  | | **p = 0.042** | |
| Currently not administered | 219 / 250 | |  | | 86.0 % | | [79.1 %; | | 90.9 %] |  | |  | |
| Physical activity and/or dietary therapy | 677 / 741 | |  | | 91.8 % | | [89.1 %; | | 93.8 %] |  | |  | |
| **Beliefs and information about diabetes** | | | | | | | | | | | | |  |
| **Low perceived risk of diabetes complications (n = 882)** |  |  | |  | |  | |  | |  | p = 0.606 | |  |
| (Fully / rather) agreement | 446 / 490 |  | | 90.7 % | | [87.1 %; | | 93.4 %] | |  |  | |  |
| (Fully / rather) disagreement | 349 / 392 |  | | 89.4 % | | [84.6 %; | | 92.8 %] | |  |  | |  |
|  |  |  | |  | |  | |  | |  |  | |  |
| **Personal control subscale (IPQ-R) (n = 938)** |  |  | |  | |  | |  | |  | p = 0.171 | |  |
| High (above median of 16) | 347 / 375 |  | | 92.8 % | | [89.1 %; | | 95.4 %] | |  |  | |  |
| Low (equal/below median of 16) | 508 / 563 |  | | 89.6 % | | [85.6 %; | | 92.5 %] | |  |  | |  |
|  |  |  | |  | |  | |  | |  |  | |  |
| **“I suppose I will have diabetes for the rest of my life” (n = 985)** |  |  | |  | |  | |  | |  | p = 0.653 | |  |
| (Fully / rather) agreement | 843 / 930 |  | | 90.1 % | | [87.3 %; | | 92.3 %] | |  |  | |  |
| Does not agree (at all) / undecided | 48 / 55 |  | | 91.9 % | | [82.1 %; | | 96.5 %] | |  |  | |  |
|  |  |  | |  | |  | |  | |  |  | |  |
| **“I consider diabetes to be a serious disease” (n = 984)** |  |  | |  | |  | |  | |  | p = 0.312 | |  |
| (Very) severe disease | 493 / 545 |  | | 89.1 % | | [85.1 %; | | 92.2 %] | |  |  | |  |
| Not / somewhat severe / no opinion | 396 / 439 |  | | 91.5 % | | [88.0 %; | | 94.0 %] | |  |  | |  |
|  |  |  | |  | |  | |  | |  |  | |  |

* The category “not employed” includes students and homemakers as well as retired or disabled respondents; Abbreviations: DMP – Disease-Management-Programme; DSME – structured diabetes self-management education; IPQ-R – Revised Illness Perception Questionnaire-subscale for control belief, CI – confidence interval, n - number
