# Supplementary material for: Diabetes self-management education programs: Results from a nationwide population-based study on characteristics of participants, rating of programs and reasons for non-participation
Source: PLoS One. 2024 Sep 12;19(9):e0310338. doi: 10.1371/journal.pone.0310338 (PMC11392325; doi:10.1371/journal.pone.0310338)
Supplement: S9 Table — * The category “not employed” includes students and homemakers as well as retired or disabled respondents. Abbreviations: DMP–Disease-Management-Programme; DSME–structured diabetes self-management education; IPQ-R–Revised Illness Perception Questionnaire-subscale for control belief. (DOCX) [file pone.0310338.s009.docx]

**S9 Table.** **Weighted logistic regression of perceived benefit of DSME (“somewhat / very helpful” vs. “not at all / rather less helpful”) on socio-demographic and disease-related characteristics, beliefs and information about diabetes (complete case analysis for n = 926)**

|  | **initial model** | | | | **final model** | | | |
| --- | --- | --- | --- | --- | --- | --- | --- | --- |
|  | **OR** | **95 % C.I.** | | **p** | **OR** | **95 % C.I.** | | **p** |
| **Socio-demographic characteristics** |  |  |  |  |  |  |  |  |
| Female (vs. male) | 0.70 | [0.35; | 1.38] | 0.299 |  |  |  |  |
| Living together with partner (vs. living alone) | 1.23 | [0.63; | 2.39] | 0.544 |  |  |  |  |
| Middle educational level (vs. low educational level) | 1.47 | [0.82; | 2.63] | 0.195 | **2.06** | **[1.10;** | **3.89]** | **0.025** |
| High educational level (vs. low educational level) | 1.10 | [0.55; | 2.20] | 0.783 | 1.55 | [0.81; | 2.99] | 0.188 |
| Employed (vs. not employed)* | 0.75 | [0.36; | 1.59] | 0.458 |  |  |  |  |
| East Germany (vs. West Germany) | 1.86 | [0.95; | 3.62] | 0.068 |  |  |  |  |
| **Disease-related factors** |  |  |  |  |  |  |  |  |
| Non-insulin medication currently administered (vs. not administered) | 1.43 | [0.80; | 2.53] | 0.224 |  |  |  |  |
| Lifestyle therapy currently administered (vs. not administered) | 1.51 | [0.83; | 2.76] | 0.180 |  |  |  |  |
| **Beliefs and information about diabetes** |  |  |  |  |  |  |  |  |
| Low control belief (IPQ-R≤16; vs. high control belief with IPQR>16) | 0.77 | [0.42; | 1.40] | 0.391 |  |  |  |  |
| Never being encouraged to attend training or group by healthcare team (vs. rarely to always) | **0.51** | **[0.28;** | **0.92]** | **0.024** | **0.46** | **[0.26;** | **0.82]** | **0.008** |
| Not familiar with DMP (vs. familiar with DMP) | 0.90 | [0.50; | 1.62] | 0.725 |  |  |  |  |
| N | 926 |  |  |  | 926 |  |  |  |
| c statistic | 0.703 |  |  |  | 0.633 |  |  |  |

* The category “not employed” includes students and homemakers as well as retired or disabled respondents

Abbreviations: DMP – Disease-Management-Programme; DSME – structured diabetes self-management education; IPQ-R – Revised Illness Perception Questionnaire-subscale for control belief
