# Supplementary material for: Diabetes self-management education programs: Results from a nationwide population-based study on characteristics of participants, rating of programs and reasons for non-participation
Source: PLoS One. 2024 Sep 12;19(9):e0310338. doi: 10.1371/journal.pone.0310338 (PMC11392325; doi:10.1371/journal.pone.0310338)
Supplement: S10 Table — * The proportion of missing information per variable ranged from 0.1% to 1.1%. For 2.1% of respondents, at least one value was imputed. Abbreviations: DMP–Disease-Management-Programme; DSME–structured diabetes self-management education; IPQ-R–Revised Illness Perception Questionnaire-subscale for control belief. (DOCX) [file pone.0310338.s010.docx]

**Table S10:** **Sensitivity analysis for weighted logistic regression of perceived benefit of DSME (“somewhat / very helpful” vs. “not at all / rather less helpful”) on socio-demographic and disease-related characteristics, beliefs and information about diabetes (n = 1002; multiple imputation by chained equations*; only final model)**

|  | **model 1** | | | |
| --- | --- | --- | --- | --- |
|  | **OR** | **95 % C.I.** | | **p** |
| **Socio-demographic characteristics** |  |  |  |  |
| Middle educational level (vs. low educational level) | **1.99** | **[1.12;** | **3.55]** | **0.020** |
| High educational level (vs. low educational level) | 1.71 | [0.91; | 3.19] | 0.094 |
| **Beliefs and information about diabetes** |  |  |  |  |
| Never being encouraged to attend training or group by treatment team (vs. rarely to always) | **0.43** | **[0.25;** | **0.73]** | **0.002** |
| n | 1002 |  |  |  |
| c statistic | 0.634 |  |  |  |

* The proportion of missing information per variable ranged from 0.1 % to 1.1%. For 2.1% of respondents, at least one value was imputed. Abbreviations: DMP – Disease-Management-Programme; DSME – structured diabetes self-management education; IPQ-R – Revised Illness Perception Questionnaire-subscale for control belief
