# Supplementary material for: Diabetes self-management education programs: Results from a nationwide population-based study on characteristics of participants, rating of programs and reasons for non-participation
Source: PLoS One. 2024 Sep 12;19(9):e0310338. doi: 10.1371/journal.pone.0310338 (PMC11392325; doi:10.1371/journal.pone.0310338)
Supplement: S11 Table — Not employed” includes students and homemakers as well as retired or disabled respondents; Abbreviations: DMP–Disease-Management-Programme; DSME–structured diabetes self-management education; IPQ-R–Revised Illness Perception Questionnaire-subscale for control belief. (DOCX) [file pone.0310338.s011.docx]

**S11 Table:** **Absolute and weighted relative frequencies of DSME-participation, stratified by DSME-format (individual counselling and group training)**

|  | Individual Counselling | | |  | Group Training | | | | |  | Test for cross-level interaction (DSME-Format* characteristics) | | |  |
| --- | --- | --- | --- | --- | --- | --- | --- | --- | --- | --- | --- | --- | --- | --- |
|  | **n /n_valid_** | **f** | |  | **n /n_valid_** | | | **f** | |  |  | | |  |
| **Overall** | 564 / 1396 | 40.8 % | |  | 833 / 1396 | | | 61.0 % | |  |  | | |  |
|  |  |  | |  |  | | |  | |  |  | | |  |
| **Age** | n = 1396 |  | |  |  | | |  | |  |  | | |  |
| 18 to 64 | 207 / 430 | 47.9 % | |  | 292 / 430 | | | 66.4 % | |  |  | | |  |
| 65 to 79 | 272 / 685 | 38.5 % | |  | 407 / 685 | | | 60.1 % | |  | p = 0.587 | | |  |
| over 80 | 85 / 281 | 26.0 % | |  | 134 / 281 | | | 47.5 % | |  | p = 0.471 | | |  |
|  |  |  | |  |  | | |  | |  |  | | |  |
| **Sex** | n = 1396 |  | |  |  | | |  | |  |  | | |  |
| Male | 320 / 719 | 45.5 % | |  | 427 / 719 | | | 61.8 % | |  |  | | |  |
| Female | 244 / 677 | 36.1 % | |  | 406 / 677 | | | 60.2 % | |  | p = 0.069 | | |  |
|  |  |  | |  |  | | |  | |  |  | | |  |
| **Family status** | n = 1394 |  | |  |  | | |  | |  |  | | |  |
| Living alone | 211 / 612 | 33.4 % | |  | 356 / 612 | | | 61.7 % | |  |  | | |  |
| Living together with partner | 352 / 782 | 46.8 % | |  | 476 / 782 | | | 60.6 % | |  | **p < 0.01** | | |  |
|  |  |  | |  |  | | |  | |  |  | | |  |
| **Educational level** | n = 1394 |  | |  |  | | |  | |  |  | | |  |
| low | 132 / 395 | 35.5 % | |  | 222 / 395 | | | 57.2 % | |  |  | | |  |
| middle | 250 / 594 | 44.3 % | |  | 374 / 594 | | | 65.8 % | |  | p = 0.985 | | |  |
| high | 181 / 405 | 48.8 % | |  | 236 / 405 | | | 60.1 % | |  | p = 0.069 | | |  |
|  |  |  | |  |  | | |  | |  |  | | |  |
| **Occupational status** | n = 1394 |  | |  |  | | |  | |  |  | | |  |
| not employed * | 431 / 1121 | 37.2 % | |  | 646 / 1121 | | | 58.7 % | |  |  | | |  |
| employed | 132 / 273 | 50.7 % | |  | 185 / 273 | | | 67.1 % | |  | p = 0.365 | | |  |
|  |  |  | |  |  | | |  | |  |  | | |  |
| **Residency** | n = 1396 |  | |  |  | | |  | |  |  | | |  |
| West Germany | 376 / 885 | 42.9 % | |  | 541 / 885 | | | 62.0 % | |  |  | | |  |
| East Germany | 188 / 511 | 36.4 % | |  | 292 / 511 | | | 59.0 % | |  | p = 0.427 | | |  |
|  |  |  | |  |  | | |  | |  |  | | |  |
| **General state of health** | n = 1394 |  | |  |  | | |  | |  |  | | |  |
| (very) good | 286 / 709 | 41.8 % | |  | 411 / 709 | | | 58.5 % | |  |  | | |  |
| Moderate to very poor | 278 / 685 | 39.9 % | |  | 421 / 685 | | | 63.3 % | |  | p = 0.108 | | |  |
|  |  |  | |  |  | | |  | |  |  | | |  |
| **Type of Diabetes** | n = 1316 |  | |  |  | | |  | |  |  | | |  |
| Type 1 diabetes | 106 / 167 | 62.7 % | |  | 131 / 167 | | | 78.2 % | |  |  | | |  |
| Type 2 diabetes | 429 / 1149 | 37.1 % | |  | 666 / 1149 | | | 59.2 % | |  | p = 0.619 | | |  |
|  |  |  | |  |  | | |  | |  |  | | |  |
| **Time since diagnosis** | n = 1388 |  | |  |  | | |  | |  |  | | |  |
| 7 years or less | 113 / 381 | 28.0 % | |  | 177 / 381 | | | 48.9 % | |  |  | | |  |
| 8 to 15 years | 183 / 469 | 40.6 % | |  | 272 / 469 | | | 62.3 % | |  | p = 0.981 | | |  |
| more than 15 years | 267 / 538 | 51.7 % | |  | 382 / 538 | | | 70.2 % | |  | p = 0.662 | | |  |
|  |  |  | |  |  | | |  | |  |  | | |  |
| **Oral antidiabetics** | n = 1396 |  | |  |  | | |  | |  |  | | |  |
| Currently not administered | 250 / 520 | 47.8 % | |  | 345 / 520 | | | 64.9 % | |  |  | | |  |
| Current therapy | 314 / 876 | 36.4 % | |  | 488 / 876 | | | 58.6 % | |  | p = 0.307 | | |  |
|  |  |  | |  |  | | |  | |  |  | | |  |
| **Insulin** | n = 1395 |  | |  |  | | |  | |  |  | | |  |
| Currently not administered | 230 / 730 | 33.1 % | |  | 352 / 730 | | | 51.7 % | |  |  | | |  |
| Current therapy | 334 / 665 | 48.5 % | |  | 480 / 665 | | | 70.1 % | |  | p = 0.446 | | |  |
|  |  |  | |  |  | | |  | |  |  | | |  |
| **Determination of insulin dosage** | n = 665 |  | |  |  | | |  | |  |  | | |  |
| Self-determination | 215 / 381 | 54.3 % | |  | 302 / 381 | | | 79.0 % | |  |  | | |  |
| Prescribed dosage | 119 / 284 | 41.8 % | |  | 178 / 284 | | | 60.0 % | |  | p = 0.153 | | |  |
| **Dietary Therapy** | n = 1393 | |  | | |  |  | |  | | |  |  | |
| Currently not administered | 226 / 577 | | 40.0 % | | |  | 332 / 577 | | 57.7 % | | |  |  | |
| Current therapy | 337 / 816 | | 41.4 % | | |  | 501 / 816 | | 63.5 % | | |  | p = 0.298 | |
|  |  | |  | | |  |  | |  | | |  |  | |
| **Physical activity or sports** | n = 1396 | |  | | |  |  | |  | | |  |  | |
| Currently not administered | 218 / 631 | | 34.8 % | | |  | 343 / 631 | | 55.6 % | | |  |  | |
| Current therapy | 346 / 765 | | 46.1 % | | |  | 490 / 765 | | 65.9 % | | |  | p = 0.850 | |
|  |  | |  | | |  |  | |  | | |  |  | |
| **“I suppose I will have diabetes for the rest of my life”** | n = 1386 | |  | | |  |  | |  | | |  |  | |
| (fully / rather) agreement | 529 / 1286 | | 41.9 % | | |  | 786 / 1286 | | 63.1 % | | |  |  | |
| Does not agree (at all) / undecided | 31 / 100 | | 29.1 % | | |  | 44 / 100 | | 42.8 % | | |  | p = 0.508 | |
|  |  | |  | | |  |  | |  | | |  |  | |
| **“I consider diabetes to be a serious disease”** | n = 1386 | |  | | |  |  | |  | | |  |  | |
| No opinion | 24 / 95 | | 24.3 % | | |  | 45 / 95 | | 43.0 % | | |  |  | |
| Not severe disease | 49 / 143 | | 36.7 % | | |  | 67 / 143 | | 48.4 % | | |  | p = 0.394 | |
| (Somewhat to very) severe disease | 485 / 1148 | | 42.6 % | | |  | 718 / 1148 | | 64.3 % | | |  | p = 0.922 | |
|  |  | |  | | |  |  | |  | | |  |  | |
| **“Are you familiar with DMP?”** | n = 1391 | |  | | |  |  | |  | | |  |  | |
| Yes | 328 / 697 | | 47.4 % | | |  | 464 / 697 | | 65.8 % | | |  |  | |
| No | 236 / 694 | | 34.5 % | | |  | 364 / 694 | | 56.0 % | | |  | p = 0.503 | |

not employed” includes students and homemakers as well as retired or disabled respondents;

Abbreviations: DMP – Disease-Management-Programme; DSME – structured diabetes self-management education; IPQ-R – Revised Illness Perception Questionnaire-subscale for control belief
